# Rapid Evolution of Virulence and Drug Resistance in the Emerging Zoonotic Pathogen *Streptococcus suis*

**DOI:** 10.1371/journal.pone.0006072

**Published:** 2009-07-15

**Authors:** Matthew T. G. Holden, Heidi Hauser, Mandy Sanders, Thi Hoa Ngo, Inna Cherevach, Ann Cronin, Ian Goodhead, Karen Mungall, Michael A. Quail, Claire Price, Ester Rabbinowitsch, Sarah Sharp, Nicholas J. Croucher, Tran Bich Chieu, Nguyen Thi Hoang Mai, To Song Diep, Nguyen Tran Chinh, Michael Kehoe, James A. Leigh, Philip N. Ward, Christopher G. Dowson, Adrian M. Whatmore, Neil Chanter, Pernille Iversen, Marcelo Gottschalk, Josh D. Slater, Hilde E. Smith, Brian G. Spratt, Jianguo Xu, Changyun Ye, Stephen Bentley, Barclay G. Barrell, Constance Schultsz, Duncan J. Maskell, Julian Parkhill

**Affiliations:** 1 The Wellcome Trust Sanger Institute, Wellcome Trust Genome Campus, Hinxton, Cambridge, United Kingdom; 2 Oxford University Clinical Research Unit, Hospital for Tropical Diseases, Ho Chi Minh City, Viet Nam; 3 Hospital for Tropical Diseases, Ho Chi Minh City, Viet Nam; 4 Institute for Cell and Molecular Biosciences, The Medical School, University of Newcastle upon Tyne, Newcastle upon Tyne, United Kingdom; 5 The School of Veterinary Medicine and Science, The University of Nottingham, Sutton Bonington Campus, Sutton Bonington, United Kingdom; 6 Nuffield Department of Clinical Laboratory Sciences, Oxford University, John Radcliffe Hospital, Headington, United Kingdom; 7 Department of Biological Sciences, University of Warwick, Coventry, United Kingdom; 8 Centre for Preventative Medicine, Animal Health Trust, Newmarket, United Kingdom; 9 Department of Biology, University of Copenhagen, Copenhagen, Denmark; 10 Groupe de recherche sur les maladies infectieuses du porc (GREMIP), Université de Montréal, Montréal, Québec, Canada; 11 Royal Veterinary College, Hatfield, United Kingdom; 12 Animal Sciences Group (ASG), Wageningen UR, UR, Lelystad, The Netherlands; 13 Department of Infectious Disease Epidemiology, Imperial College, London, United Kingdom; 14 State Key Laboratory for Infectious Disease Prevention and Control, National Institute for Communicable Disease Control and Prevention, China CDC, Beijing, China; 15 Academic Medical Center-Center for Poverty-Related Communicable Diseases, University of Amsterdam, Amsterdam, The Netherlands; 16 Department of Veterinary Medicine, University of Cambridge, Cambridge, United Kingdom; Columbia University, United States of America

## Abstract

**Background:**

*Streptococcus suis* is a zoonotic pathogen that infects pigs and can occasionally cause serious infections in humans. *S. suis* infections occur sporadically in human Europe and North America, but a recent major outbreak has been described in China with high levels of mortality. The mechanisms of *S. suis* pathogenesis in humans and pigs are poorly understood.

**Methodology/Principal Findings:**

The sequencing of whole genomes of *S. suis* isolates provides opportunities to investigate the genetic basis of infection. Here we describe whole genome sequences of three *S. suis* strains from the same lineage: one from European pigs, and two from human cases from China and Vietnam. Comparative genomic analysis was used to investigate the variability of these strains. *S. suis* is phylogenetically distinct from other *Streptococcus* species for which genome sequences are currently available. Accordingly, ∼40% of the ∼2 Mb genome is unique in comparison to other *Streptococcus* species. Finer genomic comparisons within the species showed a high level of sequence conservation; virtually all of the genome is common to the *S. suis* strains. The only exceptions are three ∼90 kb regions, present in the two isolates from humans, composed of integrative conjugative elements and transposons. Carried in these regions are coding sequences associated with drug resistance. In addition, small-scale sequence variation has generated pseudogenes in putative virulence and colonization factors.

**Conclusions/Significance:**

The genomic inventories of genetically related *S. suis* strains, isolated from distinct hosts and diseases, exhibit high levels of conservation. However, the genomes provide evidence that horizontal gene transfer has contributed to the evolution of drug resistance.

## Introduction

*Streptococcus suis* is a Gram positive coccus that colonises pigs. While it is generally carried asymptomatically in adult pigs, it can cause severe systemic disease in piglets, manifested as a rapidly fatal sepsis associated with meningitis, polyarthritis and pneumonia. Why adult pigs carry the causative bacteria asymptomatically while piglets develop acute disease is unknown. The main site of carriage in the adult are the tonsils, but bacteria have also been isolated from the nasal cavities, the gastrointestinal tract and genital tract. The carriage rate in adult pigs can approach 100% and this organism has been a worldwide problem for the pig industry for a number of years. *S. suis* has also been isolated from a range of other mammalian and avian species [1].

*S. suis* is an important zoonotic agent. The first case in humans was described in Denmark in 1968 [2]. Human infection with *S. suis* occurs sporadically in Europe and North-America and case reports suggest that it is almost exclusively related to occupational exposure to pigs or pork products. Incidences of human infection with *S. suis* are greater in S.E. Asia and China. Meningitis is the most common presentation in humans, but septicaemia and endocarditis are also seen. In areas of Vietnam, *S. suis* is the main cause of acute bacterial meningitis in adults [3] and it is the third most common cause of meningitis in Hong Kong. The incidence of *S. suis* infection in humans is almost certainly under-reported. Most cases have been described as being caused by serotype 2 isolates, but other serotypes can cause human disease [4].

Recently two outbreaks of severe acute disease in humans with high morbidity and mortality in humans have been reported in China [5], in both cases due to serotype 2 strains. An outbreak in 1998 killed 14 of 25 patients, and an outbreak in 2005 affected 204 people, killing 38 of them (19%), a mortality rate around two to four times that previously reported. A high proportion of people in these outbreaks had a toxic shock-like syndrome, and most of the deaths occurred in this group rather than in those suffering meningitis. Streptococcal toxic shock syndrome (STSS) was previously associated with *Streptococcus pyogenes*, so the emergence of STSS due to presumptive zoonotic *S. suis* infection is of considerable concern [6]. One isolate from each of these outbreaks has been sequenced [7] and this has identified a proposed pathogenicity island (PI) that may be involved in this particular clinical manifestation of *S. suis* infection, although this remains speculative at this stage. The PI was identified through comparison of the Chinese strains to the unfinished, unannotated genome sequence of strain P1/7 from the Sanger Institute. In this paper we present the completed fully annotated genome sequence of strain P1/7. In addition we have sequenced the genomes of two other *S. suis* strains, SC84 and BM407, which are human isolates from China and Vietnam respectively. Strain SC84 is a representative of the of 2005 outbreak in China [8].

## Materials and Methods

### Bacterial strains, growth and DNA isolation

*S. suis* strain P1/7 was isolated from an ante-mortem blood culture from a pig dying with meningitis [9], and is ST1 by MLST [10]. *S. suis* strain BM407 is also ST1, and was isolated from CSF from a human case of meningitis in Ho Chi Minh City, Vietnam in 2004 [3]. *S. suis* strain SC84 is ST7, which is closely related to ST1, and was isolated from a case of streptococcal toxic shock-like syndrome in Sichuan Province, China in 2005 [8]. Strain P1/7 is resistant to gentamycin, streptomycin, neomycin, nalidixic acid, and sulfamethoxazole, and sensitive to penicillin, ampicillin, cephalotin, erythromycin, tulathromycin, clarythromycin, lincomycin, clindamycin, pirlimicin, tetracycline, trimethoprim-sulfa, ciprofloxacin, and chloramphenicol. Strain BM407 is resistant to trimethoprim-sulfamethoxazole, tetracycline, erythromycin, azithromycin and chloramphenicol and susceptible to penicillin, ceftriaxone and vancomycin. Strain SC84 is resistant to tetracycline, and susceptible to penicillin, ampicillin, cefotaxime, ceftriaxone, cefepime, meropenem, levofloxacin, chloramphenicol, erythromycin, azithromycin, clindamycin, and vancomycin [11].

Bacteria were cultured in Todd-Hewitt-broth at 37°C for 18 h and pelleted at 10,000×g. The cells were resuspended in 30 ml of lysis solution (10 mM NaCl, 20 mM Tris HCl pH 8, 1 mM EDTA, 0.5% SDS) and incubated at 50°C overnight. Three ml of 5 M sodium perchlorate was added and incubated for 1 h at ambient temperature. After phenol chloroform extraction the DNA was precipitated with ethanol, spooled into deionised water and stored at −20°C. DNA was also extracted using a genomic DNA extraction kit (G-500, Qiagen).

### Whole genome sequencing

The genome of *S. suis* strain P1/7 was sequenced to approximately 8-fold coverage, from pUC18 (insert size 2.8–3.3 kb), and pMAQ1b_SmaI (insert size 3.0–3.3 kb) genomic shotgun libraries using big-dye terminator chemistry on ABI3730 automated sequencers. End sequences from large insert BAC libraries in pBACehr (insert size 10–25 kb) and pBACe3.6 (insert size 12–15 kb) were used as a scaffold. All repeat regions were bridged by read-pairs or end-sequenced polymerase chain reaction (PCR) products.

*S. suis* SC84 and BM407 genomes were sequenced using multiple sequencing technologies to generate contiguous drafts. The bulk of the sequencing for both projects was generated using 454/Roche GS20 and Solexa sequencing platforms. The 454 sequencing of strain SC84 generated 399,145 reads with an average length of 102.7 bp, which assembled *de novo* into 281 non-redundant contigs using the 454/Roche Newbler assembly program. The 454 sequencing of strain BM407 generated 223,282 reads with an average length of 103.1 bp, which assembled *de novo* into 1939 non-redundant contigs. The Solexa sequencing of strain SC84 generated 32,016,513 reads with a length of 37 bp, and of strain BM407 generated 1,769,043 reads with a length of 37 bp which were each assembled by alignment to a reference (strain P1/7) sequence using the ssaha2 [12] alignment program. Data from the 2 technologies, were merged to produce 39 non-redundant contigs in SC84, and 390 non-redundant contigs in BM407.

The merging process involved using the random *in silico* sheering of the 454 *de novo* assembly consensus contigs to create 500 bp consensus reads with 250 bp overlaps. This generated a fasta tiling path representing each of the 454 *de novo* assembly contigs. These were then assembled together with all the capillary shotgun reads using phrap2gap [13] to merge the two technologies

The contigs were reordered based on BLAST [14] alignments with strain P1/7. The gaps between these contigs were closed by directed PCR and the products sequenced with big dye terminator chemistry on ABI3730 capillary sequencers. Aligned Solexa sequencing was merged to confirm assembly and also confirm sequence in 454 only regions. For the SC84 project capillary coverage was 5×, 454 coverage was 19× and Solexa coverage was 190×, and for the BM407 project capillary coverage was 5×, 454 coverage was 11× and Solexa coverage was 20×.

The sequences and annotations of the *Streptococcus suis* P1/7, SC84 and BM407 genomes have been deposited in the EMBL database under accession numbers AM946016, FM252031, and FM252032 and FM252033 respectively.

The sequence was annotated using Artemis software [15]. Initial coding sequence (CDS) predictions were performed using Orpheus [16], Glimmer2 [17], and EasyGene [18] software. These predictions were amalgamated, and codon usage, positional base preference methods and comparisons to the non redundant protein databases using BLAST [14] and FASTA [19] software were used to refine the predictions. The entire DNA sequence was also compared in all six potential reading frames against UniProt, using BLASTX [14] to identify any possible coding sequences previously missed. Protein motifs were identified using Pfam [20] and Prosite [21], transmembrane domains were identified with TMHMM [22], and signal sequences were identified with SignalP version 2.0 [23]. rRNAs were identified using BLASTN [14] alignment to defined rRNAs from the EMBL nucleotide database; tRNAs were identified using tRNAscan-SE [24]; stable RNAs were identified using Rfam [25].

### Comparative genomics

Comparison of the genome sequences was facilitated by using the Artemis Comparison Tool (ACT) [26] which enabled the visualization of BLASTN and TBLASTX comparisons [14] between the genomes. Orthologous proteins were identified as reciprocal best matches using FASTA [19] with subsequent manual curation. Pseudogenes had one or more mutations that would prevent correct translation; each of the inactivating mutations was subsequently checked against the original sequencing data. Small scale variation including: SNPs and insertions and deletions, were identified by using the SNP detection pipeline of MUMmer [27]. Clonalframe v1.1 was used to identify regions of the *S. suis* chromosomes associated with recombination [28].

*Streptococcus* sequences used for comparative genomic analysis were: *S. suis* 05ZYH33 (accession number CP000407) [7], *S. suis* 98HAH33 (accession number CP000408) [7], *S. pyogenes* Manfredo (accession number AM295007) [29], *Streptococcus equi* 4047 (accession number FM204883) [30], *Streptococcus uberis* 0140J (accession number AM946015) [31], *Streptococcus thermophilus* CNRZ1066 (accession number CP000024) [32], *Streptococcus pneumoniae* TIGR4 (accession number AE005672) [33], *Streptococcus sanguinis* SK36 (accession number CP000387) [34], *Streptococcus mutans* UA159 (accession number AE014133) [35], *Streptococcus agalactiae* NEM316 (accession number AL732656) [36], and *Streptococcus gordonii* str. Challis substr. CH1 (accession number CP000725) [37]. The sequences were also compared wth *Lactococcus lactis* subsp. *lactis* IL1403 (accession number AE005176) [38].

### Phylogenetic analysis

Sequences alignment were made using R-coffee [39] and the alignments inspected and edited using Seaview (v4.0) [40]. Bayesian analysis was performed using Mr Bayes [41], [42]. The model of sequence evolution used was the generalized time-reversible (GTR) model with gamma-distributed rate variation. The analysis was run with 2×10^5^ generations, and the first 5×10^4^ were discarded as burn-in. The tree drawn with FigTree (v1.2.2; http://tree.bio.ed.ac.uk/software/figtree/).

## Results

### The genome of *S. suis* strain P1/7, a pig disease isolate

**Figure 1:**
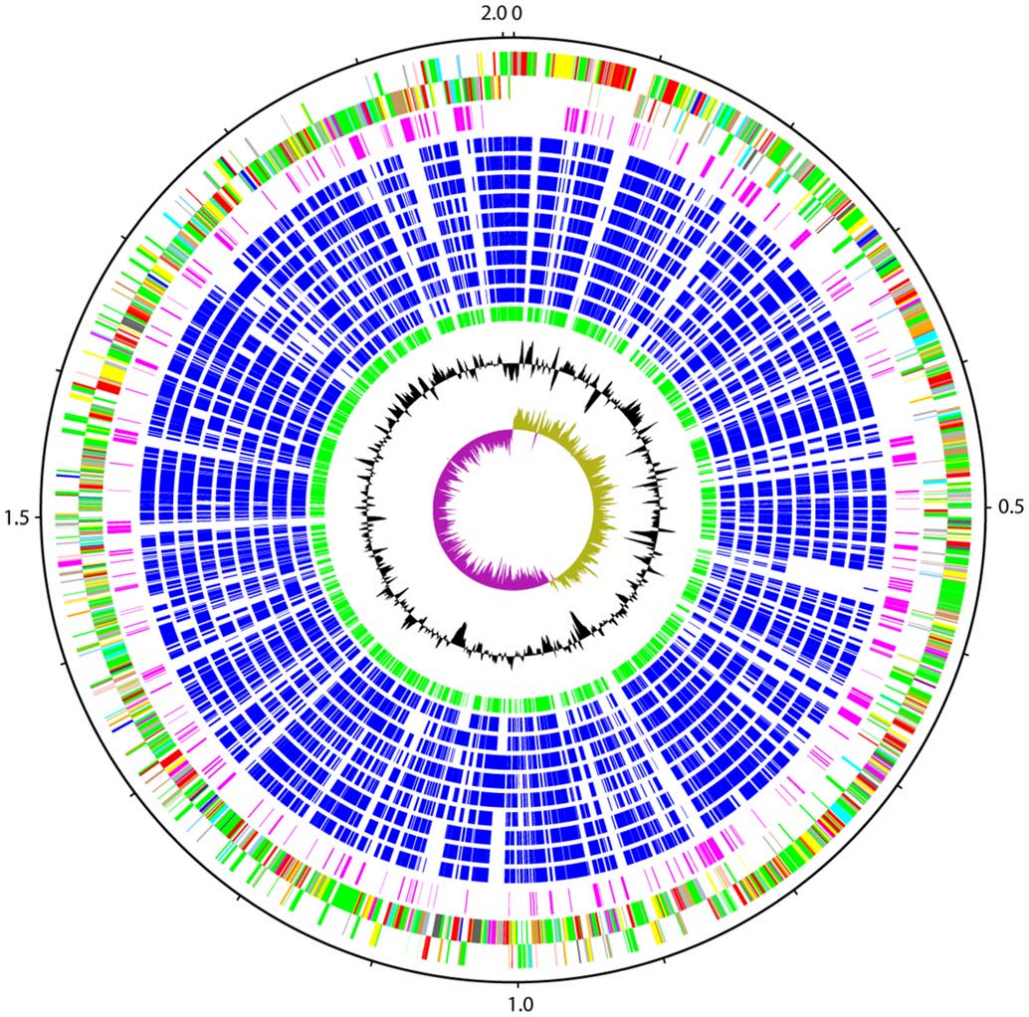
Schematic circular diagram of the *S. suis* P1/7 genome. Key for the circular diagram (outer to inner): scale (in Mb); annotated CDSs coloured according to predicted function are shown on a pair of concentric circles, representing both coding strands; *Streptococcus suis* orphan CDSs, purple; orthologue matches shared with the Streptococcal species, *S. mutans* UA159, *S. gordonii* Challis CH1, *S. sanguinis* SK36, *S. pyogenes* Manfredo, *S. equi* 4047, *S. agalactiae* NEM316, *S. uberis* 0140J, *S. pneumoniae* TIGR4, *S. thermophilus* CNRZ1066, blue; orthologue matches shared with *Lactococcus lactis* subsp. *lactis*, green; G+C% content plot; G+C deviation plot (>0% olive, <0% purple). Colour coding for P1/7 CDS functions: dark blue, pathogenicity/adaptation; black, energy metabolism; red, information transfer; dark green, surface associated; cyan, degradation of large molecules; magenta, degradation of small molecules; yellow, central/intermediary metabolism; pale green, unknown; pale blue, regulators; orange, conserved hypothetical; brown, pseudogenes; pink, phage and IS elements; grey, miscellaneous.

**Figure 2:**
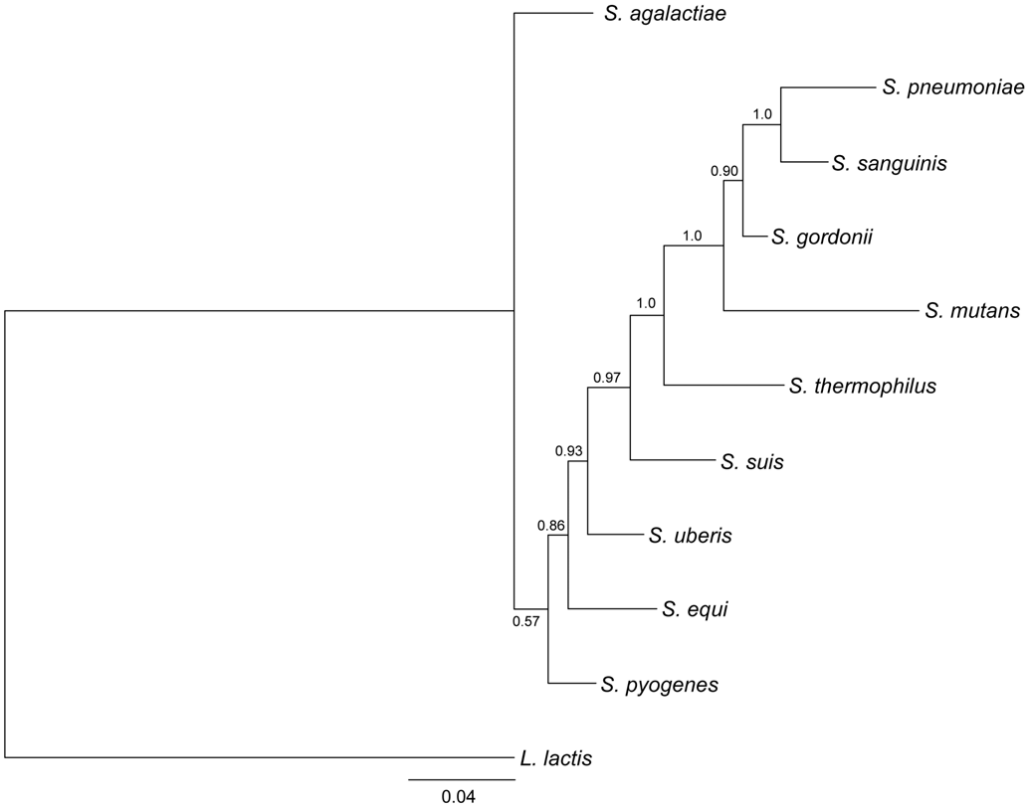
Phylogenetic relationships of *S. suis* to the other genome sequenced streptococci. Unrooted majority-rule tree of Bayesian analysis of combined 16S rRNA and RNase P RNA gene datasets. The was tree built from 16S rRNA and *rnpB* gene sequences from the genomes of the *Streptococcus* species: *S. suis* P1/7, *S. mutans* UA159, *S. gordonii* Challis CH1, *S. sanguinis* SK36, *S. pyogenes* Manfredo, *S. equi* 4047, *S. agalactiae* NEM316, *S. uberis* 0140J, *S. pneumoniae* TIGR4, and *S. thermophilus* CNRZ1066. *Lactococcus lactis* subsp. *lactis* IL1403 was included as an outgroup. The numbers at the branches are posterior probabilities indicating the support for the branch. The bar indicates the number of substitutions per site (4 per 100 sites).

The genome of *Streptococcus suis* P1/7 consists of a single circular chromosome of 2,007,491 bp (Figure 1; accession number AM946016) containing 1,908 predicted protein coding sequences (CDSs; Table 1), 82 of which are pseudogenes or gene fragments (Table S1). *S. suis* is phylogenetically distinct among the streptococci for which genome sequences are available (Figure 2). Pairwise genomic comparisons reveal very little conservation of gene order with other species (data not shown). Orthologue comparisons against all of the other available streptococcal genomes identified that approximately 60% of the P1/7 genome is orthologous to CDSs in the genomes of other *Streptococcus* species (Table 2). The highest number of orthologous matches was identified in *S. sanguinis* (66.1%), followed by *S. gordonii* (65.6%).

**Table 1:** General properties of the genomes of *S. suis* strains P1/7, SC84 and BM407.

	P1/7	SC84	BM407
**Size (bp)**	2,007,491	2,095,898	2,146,229
**G+C content**	41.3%	41.1%	41.1%
**CDSs**	1908	1985	2040
**Coding %**	85.1%	84.8%	83.9%
**Av. gene length (bp)**	931	933	932
**rRNA (16S-23S-5S)**	4	4	4
**tRNA**	56	56	56
**miscellaneous RNA**	13	13	13
**Pseudogenes and partial genes**	82	87	108
**IS elements**	27	28	32
**Genomic islands**	4	4	3
**ICE regions**	0	1	2
**Plasmid**	-	-	1*

**Table 2:** Orthologues of *S. suis* P1/7 CDSs in other streptococci.

	% of CDSs with orthologous matches
*S. sanguinis* SK36	66.1
*S. gordonii* CH1	65.6
*S. agalactiae* nem316	64.4
*S. agalactiae* 2603v	64.0
*S. agalactiae* A909	63.6
*S. uberis* 0140J	63.9
*S. pneumoniae* D39	64.2
*S. pneumoniae* TIGR4	63.8
*S. pneumoniae* R6	63.6
*S. mutans* UA159	61.2
*S. equi* 4047	60.1
*S. pyogenes* Manfredo	57.9
*S. thermophilus* CNRZ1066	55.6
*S. thermophilus* LMG 18311	55.1

Orthologues matches are displayed as a percentage of the total CDSs in the P1/7 genome.

**Figure 3:**
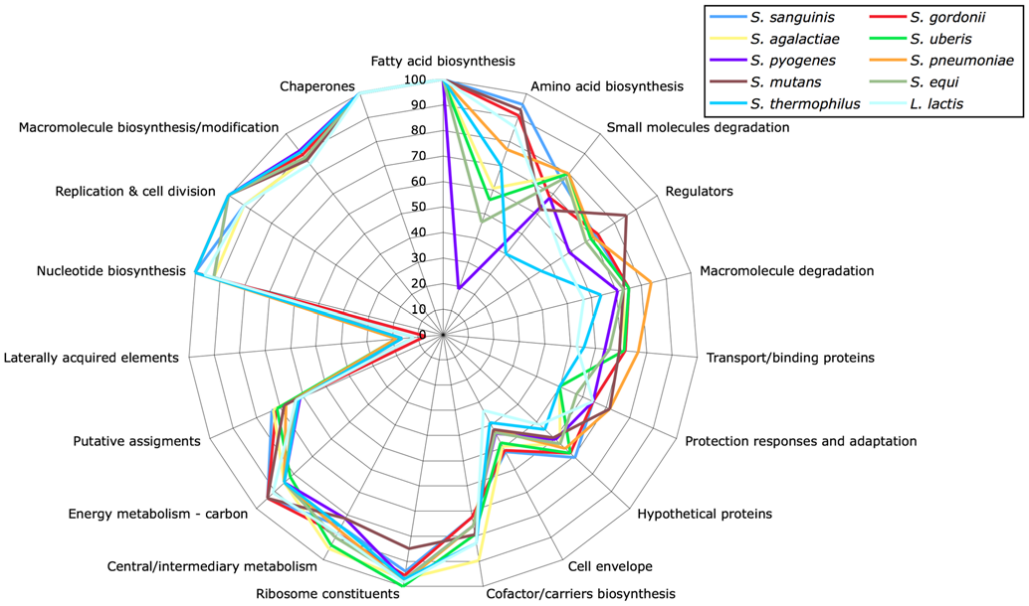
Functional distribution of Streptococcal orthologues in the *S. suis* P1/7 genome. Functional groups are displayed clockwise on the web graph, in decreasing range of orthologue matches for a single category.

Analysis of the functions of the P1/7 orthologues present in other streptococcal species identified that some functional groups are more widely conserved than others (Figure 3), and that some exhibit a wide range of distribution across the species compared. Many of the core housekeeping functions such as fatty acid metabolism, chaperones, macromolecule biosynthesis, and nucleotide biosynthesis exhibit the highest conservation. For these classes, >85% of the CDSs of these classes have orthologous matches in all the other streptococci compared. In some cases, for example ribosomal proteins, a lower level of orthologous matches in a strain is due to differences in gene prediction, rather than the absence of a CDS *per se*. In contrast, some functional groups such as cofactor biosynthesis and amino acid biosynthesis exhibit a lower level of conservation and probably reflect the available nutrients in the niches occupied by the individual streptococcal species. Many of the more variably distributed orthologous functional groups are associated with the acquisition and uptake of nutrients, for example macromolecule and small molecule degradation, as well as responses to environmental stresses and stimuli.

Excluding CDSs in mobile genetic elements (MGE), 283 CDSs (14.9%) in the P1/7 genome do not have orthologous matches (have been) in any of the other *Streptococcus* species (Table S2). These have been designated *S. suis* orphans and include: a transporter cluster (SSU0039 to SSU0044), a putative endopeptidase (SSU0152), a glucosamine-6-phosphate isomerase (SSU0206), a putative lipase (SSU0304), a putative deoxyguanosinetriphosphate triphosphohydrolase (SSU0327), capsule associated CDSs (SSU0520 to SSU0529), a UDP-galactopyranose mutase (SSU0563), a MATE family transporter (SSU0573), EPS associated CDSs (SSU1115, SSU1125 and SSU1128), a putative oligopeptidase (SSU1679), and a putative choline binding protein (SSU1911) (Table S2).

### Virulence genes in the P1/7 genome

The mechanisms by which *S. suis* cause disease in humans and pigs are poorly understood. Many putative virulence functions have been identified, however no single function is crucial for the pathogenesis of *S. suis*. Many putative virulence genes are only present, or expressed, in some *S. suis* isolates, suggesting that combinations of virulence factors may be more important than individual functions. Additionally, several of the functions may be important for colonization and carriage, rather than disease. Analysis of the *S. suis* genomes reveals an array of CDSs encoding potentially important functions.

#### Polysaccharides and capsule

Like most other pathogens that can cause meningitis, *S. suis* expresses a polysaccharide capsule. P1/7 is serotype 2 and contains a locus (SSU0515 to SSU0529) for the biosynthesis of type 2 capsule [43], [44]. The structure of the *S. suis* serotype 2 capsule is unknown, but it is composed of glucose, galactose, N-acetylglucosamine, rhamnose and sialic acid in a ratio of 1∶3∶1∶1∶1 [45]. Although 35 serotypes of *S. suis* have been described, type 2 is most frequently associated with disease in pigs and humans [3], [46]–[48]. Capsule plays a role in the virulence of *S. suis*; type 2 capsule mutants are avirulent in murine and pig models of infections [43], [49] The capsule itself can mediate prostaglandin E2 (PGE2) and matrix metalloproteinase 9 (MMP-9) production by human macrophages [50] suggesting a role in pathogenesis of disease.

The P1/7 genome also contains a cluster of genes that appears to encode the components required for the biosynthesis of a putative exopolysaccharide (SSU1111 to SSU1133; Figure S1a). Included in this are CDSs encoding enzymes for synthesis of the exopolysaccharide building blocks, for processing and for export. Analysis of the cluster suggests that it is involved in the synthesis of a rhamnose-glucose-like polysaccharide (RGP), and is similar to components of RGP biosynthesis identified in *S. mutans* (Figure S1b). Four loci have been identified in *S. mutans* that encode proteins necessary for RGP synthesis: RmlA, RmlC, RmlB, and RmlD direct the synthesis of dTDP-L-rhamnose from D-glucose-1-phosphate [51], [52]; GluA produces UDP-D-glucose [53], RgpG initiates RGP synthesis by transfer of N-acetylglucosamine-1-phosphate to a lipid carrier [54], and proteins from an operon encoding six proteins (*rgpABCDEFHI*) are required for the transport and assembly of the rhamnan repeat unit backbone, and glucose side chain formation [55], [56].

The putative exopolysaccharide biosynthetic cluster between CDSs SSU1111 and SSU1133, contains homologues of *rmlACB* and *rmlD* (SSU1129, SSU1130, SSU1132 and SSU1133), and *rgpABCD* and *F* (SSU1124, SSU1123, SSU1122, SSU1121 and SSU1120) (Figure S1a). The two clusters of *rml* and *rgp* homologues are separated by four CDSs encoding a putative sulphatase (SSU1125), a mannosyl-glycoprotein endo-beta-N-acetylglucosaminidase gene fragment (SSU1126), a putative N-acetylmuramoyl-L-alanine amidase (SSU1127), and a surface-anchored protein containing an LPXTS motif (SSU1128). In addition to the homologues of the *S. mutans rpg* genes, there is also a flippase (SSU1119), and six glycosyl transferases (SSU1117, SSU1116, SSU1115, SSU1114, SSU1113 and SSU1111). Homologues of *gluA* (SSU1828) and *rgpG* (SSU1672) are located at two other loci on the P1/7 genome. Unlike the *S. mutans* RGP cluster, the P1/7 cluster does not contain homologues of *rmlE*, *rmlH* and *rmlI*. These genes encode proteins that are involved in glucose side chain formation [56], therefore it is likely that the polysaccharide produced by P1/7 has a different repeat unit structure to that produced by *S. mutans*.

The cluster does not appear to encode an obvious repeat unit polymerase. Downstream of the flippase is a membrane protein (SSU1118) that has weak similarity matches to proteins of unknown function. Due to the sequence diversity of repeat unit polymerases, it is not always possible to annotate them by sequence similarity. Hydrophobicity plot profile comparison has been used to identify tentatively these polymerases [57]. A comparison of the hydrophobicity plot of SSU1118 with those of known polymerases identified a similar profile (data not shown).

RGPs constitute serospecific-antigens that form the basis of some streptococcal serotyping. These molecules are associated with modulating host immune cell interactions, and may have a role in eliciting sepsis. Streptococcal RGPs stimulate human monocytes *in vivo* to release inflammatory cytokines, including tumor necrosis factor alpha and interleukin-1β [58], induce NO synthase activity [59], and confer resistance to phagocytosis by human polymorphonuclear leukocytes [60]. Elevated NO production has been observed in animal models of bacterial meningitis and also in human patients with the disease [61], [62]. In a mouse model of *S. suis* infection, high levels of inflammatory cytokines were observed both systemically and in the central nervous system [63]. Distinct from the type 2 capsule, it is possible that the exopolysaccharide encoded by this cluster may have an important role in virulence and may contribute to the inflammatory response during *S. suis* infection.

#### Protein secretion and secreted proteins

In the publication by Chen *et al.* describing the genomes of two Chinese *S. suis* isolates [7], they presented possible virulence-associated factors or pathways in *S. suis*, including strain P1/7. This analysis identified thirteen components of a Type II secretion system and one component of a Type III secretion system in the genome of strain P1/7. Our analysis of the genome failed to identify components of either secretion system, although components of Sec and SRP pathways were identified. Neither Type II, nor Type III secretion systems have been identified in Gram-positive bacteria, and it is therefore very unlikely that *S. suis* secretes proteins via these pathways. It would appear that the description of these secretion system components in *S. suis* is a case of mistaken identity due to sub-optimal transitive annotation. The *S. suis* genome contains CDSs that have weak similarity to components of these Gram-negative protein secretion systems, but these genes in *S. suis* encode proteins that are involved in the transport of other molecules, or are components of functionally distinct systems. The observed similarity owes more to distant evolutionary relationships rather than conservation of function [64].

Suilysin (SSU1231) is a secreted thiol-activated cytolysin [65] produced by some strains of *S. suis* [66]. This protein is part of a family of cytolysins that play a role in disease caused by several bacterial pathogens [67]. The role that suilysin plays in *S. suis* disease is unclear. Immunization of pigs with suilysin provided partial protection from *S. suis* infection [68]. However, while a suilysin mutant was avirulent in a mouse infection model, it was only slightly attenuated in a model of infection in pigs [69].

#### Surface proteins

Several of the previously characterized *S. suis* virulence factors are cell wall-anchored proteins. These sortase-processed proteins contain a C-terminal LPXTG-type processing motif. The genome of strain P1/7 contains a large number (28) of proteins of this type: all but five contained C-terminal LPXTG motifs followed by non-polar and basic amino acid motifs. The other five putative surface proteins (SSU1128, SSU1885, SSU1186, SSU1888 and SSU1889) contained LPXTS, IPXTG, IPXTG, YPXTG and LYXTG motifs respectively. Previous studies have identified five sortase homologues in *S. suis* (*srtA*-*E*) by PCR screening with degenerate primers [70]. An additional sortase CDS was identified (SSU0428) in the P1/7 genome [71].

In addition, the *S. suis* P/7 genome contains a protein (SSU0879) with an atypical N-terminal LPXTG-type motif. This protein is similar to the large surface zinc metalloproteinases of *S. pneumoniae*; 39.9% identical to the zinc metalloprotease precursor ZmpC [72], 33.7% identical to Immunoglobulin A (IgA)1 protease precursor Iga [73], and 28.3% identical to the zinc metalloprotease precursor ZmpB [74]. In *S. pneumoniae* these proteins interact directly with the host immune system, and play an inferred role in virulence. ZmpC mutants in *S. pneumoniae* led to reduced mortality in an experimental mouse model of intranasal challenge and sepsis [72]. ZmpC cleaves human matrix metalloproteinase 9 (MMP-9), thought to lead to the proteolytic activation of this matrix remodelling protein [72]. Matrix metalloproteinases can contribute to blood–brain barrier (BBB) disruption during bacterial meningitis [75], [76]. MMP-9 produced by leukocytes during meningitis is essential to enable them to migrate through the BBB. A consequence of the increase in the permeability of the BBB mediated by MMP-9, is that bacterial cells are able to migrate to the central nervous system and cause further tissue damage. *S. suis* capsule induces MMP-9 production in macrophages in an *in vivo* model [50]: it is possible that the *S. suis* zinc metalloproteinase may also play a role in increasing MMP-9 activity.

IgA1 protease mediates the site-specific cleavage of human IgA1 in the hinge region [73]. IgA is the major class of immunoglobulin in mucosal secretions of the upper respiratory tract, and functions by interfering with microbial attachment to host tissues. The activity of IgA1 protease may help pathogens to subvert the antigen specificity of the humoral immune response, and facilitate adhesion and persistence at the mucosal surface [77].

Analysis of extracellular proteins produced by *S. suis* identified two proteins, muramidase-released protein (MRP) [78] and extracellular protein factor (EF) [79], associated with disease and mortality in pigs (MRP^+^, EF^+^) [79]. Variation in the expression of these proteins has been observed in *S. suis* isolates. Although MRP and EF were originally identified as markers of disease, most *S. suis* serotype 2 strains isolated from diseased pigs in Canada are phenotypically negative for MRP and EF [80]. Additionally, mutation of both proteins had no measurable effect on pathogenicity [81]. P1/7 is phenotypically positive for MRP and EF (MRP^+^, EF+). In the genome of P1/7, EF appears to be encoded in the first ORF of a larger mutated CDS (SSU0171). The entire CDS encodes a putative surface-anchored protein, and is split into three reading frames by a frameshift and nonsense mutations; the 5-prime reading frame encoding EF, contains an N-terminal signal sequence, and the 3-prime reading frame contains a LPXTG sortase-processing motif. The EF+ phenotype of P1/7 is probably due to the expression of the 5-prime reading frame.

Unlike MRF and EF, opacity factor (OFS) is a large surface-associated protein that may play a role in the virulence of *S. suis* [82]. The N-terminus of OFS is homologous to the N-terminal regions of the fibronectin-binding protein A (FnBA) of *Streptococcus dysgalactiae* and the serum opacity factor of *S. pyogenes*. Mutational studies have suggested a role for OFS in virulence, but not colonization. However in strain P1/7 OFS (SSU1474) is mutated, containing two frameshift mutations.

Two of the LPXTG proteins were found in a cluster similar to the pilus islands identified in *S. agalactiae* [83], and comprise the main pilus subunit (SSU0427) and ancillary subunit of this pilus (SSU0425). In addition the cluster contains a signal peptidase (SSU0424) and a sortase (SSU0428). The genome contains a second pilus cluster, containing 3 sortases, *srtD*, *srtC*, *srtB* (SSU1881, SSU1882, SSU1883), ancillary protein 1 (SSU1885), the main pilus subunit (SSU1886), ancillary protein 2 (SSU1888) and ancillary protein 3 (SSU1889). *In silico* analysis of the P1/7 genome suggests that this strain does not express complete pili, as both of the clusters contain pseudogenes; SSU0425 contains a nonsense mutation, and SSU1886 and SSU1888 contain frameshift mutations. To this end it is worth noting that orthologous CDS in the other sequenced *S. suis* strains are also pseudogenes. The genome also contains an incomplete pilus cluster that contains a signal peptidase (SSU0450) and sortase SrtE (SSU0453), but lacks pilus subunits and ancillary subunits. Between the signal peptidase and sortase there are a putative exported protein (SSU0451) and a transposase fragment (SSU0452). It is possible that the putative exported protein represents the N-terminal region of a pilus protein that has been truncated by a deletion event that generated the partial transposase sequence. Evidence for the potential role of pili in *S. suis* pathogenesis has come from *in vitro* work looking for preferentially expressed genes in *S. suis* upon interaction with porcine brain microvascular endothelial cells [71]. The signal peptidase (SSU0424) of the *S. agalactiae*-like cluster is upregulated, as is *srtE*, the sortase belonging to the incomplete pilus cluster.

Comparison of cell wall-anchored proteins among streptococcal species shows that they are one of the most variable components of the genome; 13 out of the 29 proteins in P1/7 were unique to *S. suis* (Table S2), the rest being intermittently distributed. Interestingly, orthologues of several of the *S. suis* cell wall-anchored proteins, or proteins associated with their processing, were conserved within the meningitis-associated streptococci (Table 2). Six of the cell wall-anchored proteins, and four of the sortases were found in *S. agalactiae* and/or *S. pneumoniae*, and were absent in other streptococci [84].

In addition to LPXTG-type cell wall-anchored proteins, the P1/7 genome also contains other surface-exposed proteins that may play an important role in modulating host-cell interactions. The P1/7 genome encodes a protein, SSU0496, which contains a Mac 1 domain (PF09028), an N-terminal signal sequence and a C-terminal transmembrane domain. The Mac 1 domain is found in a small number of streptococcal proteins that modulate the immune response. Mac, also known as IdeS, is a cysteine protease secreted by *S. pyogenes* [85], [86]. Two allelic families of Mac have been identified in *S. pyogenes*; Mac I and Mac II [87] exhibit sequence divergence in the middle third of the proteins (∼50% amino acid identity in this region). Mac was originally named because it had limited sequence homology to the α-subunit of the leukocyte β2 integrin, Mac-1 [85]. *S. pyogene*s Mac proteins block polymorphonuclear leukocyte opsonophagocytosis and inhibit the production of reactive oxygen species [87]. The mechanisms of action of the *S. pyogenes* Mac allelic families differ; Mac I binds to, and cleaves, the hinge region of human IgG [88] whereas Mac-2 exhibits lower levels of endopeptidase activity, and does not bind IgG, but instead to the IgG receptor, Fcγ [89]. Homologues of Mac have been identified in *Streptococcus equi* subsp. *equi* (349 amino acids), the causative agent of strangles, and the related pathogen *Streptococcus equi* subsp. *zooepidemicus* [90]. In *S. equi* the Mac homologue is expressed during infection, and reduces the antiphagocytic activity of equine neutrophils [91].

SSU0496 is similar (∼29% sequence identity) in the N-terminal region to the *S. pyogenes* Mac I and II proteins. In comparison, the *S. suis* protein is considerably larger than the *S. pyogenes* Mac proteins, comprising 1141 amino acids as opposed to ∼340 amino acids. The enzymatic activities of *S. pyogenes* Mac proteins have been characterized, and they belong to a novel family of cysteine proteases [88]. Protein alignment of the *S. suis* protein with the *S. pyogenes* proteins shows that the amino acids of the catalytic triad (Cys94, His262 and Asp284) are conserved. The *S. suis* protein however, is missing an Arg-Gly-Asp (RGD) integrin-binding motif present in the other streptococcal proteins, suggesting that while it may possess the peptidase activity of its homologues it may lack the ability to bind integrin.

### Comparative genomics of streptococci that can cause meningitis

Several species of *Streptococcus* have been associated with meningitis in humans: *S. pneumoniae* is an important cause of bacterial meningitis [92], and *S. agalactiae* is important in this regard [93]. Comparative genomic analysis was used to identify components of the *S. suis* genome shared with *S. pneumoniae* and *S. agalactiae* that were absent in other streptococci. Three strains of *S. agalactiae*, NEM316 [94], 2603V/R [95] and A909 [96] and three strains of *S. pneumoniae*, R6 [97], TIGR4 [33] and D39 [98], have been sequenced thus far. Whilst *S. pneumoniae* strain TIGR4 is from a case of invasive disease, and *S. agalactiae* strains NEM316, 2603V/R and A909 are all clinical isolates, none of the strains appears to come from a meningitis case. 71 CDSs were identified that were present in at least one of the strains of *S. pneumoniae* or *S. agalactiae*, but were absent in other *Streptococcus* species (Table 3). Of these 12 were found in MGEs in the P1/7 genome. The distribution of orthologue matches is varied between species and between strains; only 5 CDSs (SSU0685, SSU0686, SSU0687, SSU1050 and SSU1403) have orthologue matches in all of the strains for both species. Amongst these common CDSs are 3 CDS that form part of an operon and encode a phosphomethylpyrimidine kinase, a hydroxyethylthiazole kinase and a thiamine-phosphate pyrophosphorylase. These 3 CDSs catalyse the final steps in the biosynthesis of thiamine monophosphate from pyrimidine and thiazole molecules. The other CDSs in this operon, SSU0684, encodes a putative phosphatase which does not have any orthologue matches. Another of the CDSs common to *S. suis*, *S. agalactiae* and *S. pneumoniae* encodes a PadR family regulatory protein

**Table 3:** CDSs in the genome of *S. suis* strain P1/7 that have orthologues in *S. pneumoniae* and/or *S. agalactiae*, but not other streptococci.

ID	Product	*S. agalactiae*			*S. pneumoniae*		
		2603V/R	A909	NEM316	TIGR4	D39	R6
SSU0003	diacylglycerol kinase protein	+	+	+	−	−	−
SSU0103*	replication initiation factor	+	+	−	−	−	−
SSU0104*	conserved hypothetical protein	+	+	−	−	−	−
SSU0106*	FtsK/SpoIIIE family protein	+	+	−	−	−	−
SSU0110	conserved hypothetical protein	−	−	+	−	−	−
SSU0173	putative membrane protein	+	+	+	−	−	−
SSU0174	ABC transporter ATP-binding protein	+	+	+	−	−	−
SSU0200	conserved hypothetical protein	−	−	−	+	+	+
SSU0226	putative transcriptional regulator	−	−	−	+	+	+
SSU0228	putative membrane protein	−	−	−	+	+	+
SSU0415	Fic protein family	−	−	−	+	+	+
SSU0417	conserved hypothetical protein	−	−	−	+	+	+
SSU0418	putative DNA-binding protein	−	−	−	+	+	+
SSU0423	hypothetical protein	−	+	−	−	−	−
SSU0424	putative signal peptidase I 2	−	+	−	−	−	−
SSU0428	sortase	−	+	−	−	−	−
SSU0440	acetyltransferase (GNAT) family protein	−	−	−	+	+	+
SSU0452*	putative transposase (fragment)	−	−	−	+	−	−
SSU0520	putative rhamnosyl transferase	−	−	−	−	+	−
SSU0524	oligosaccharide repeat unit polymerase	+	−	−	−	−	−
SSU0533	putative lipooligosaccharide sialyltransferase	+	+	+	−	−	−
SSU0535	putative N-acetylneuraminic acid synthase	+	+	+	−	−	−
SSU0537	putative transferase	+	+	+	−	+	+
SSU0538	N-acylneuraminate cytidylyltransferase	+	+	+	−	−	−
SSU0541*	putative transposase	−	−	−	−	+	−
SSU0544*	putative transposase	−	−	−	+	−	+
SSU0549*	putative transposase (fragment)	−	+	−	−	−	−
SSU0566*	putative transposase	−	−	−	−	−	+
SSU0601	conserved hypothetical protein	+	+	+	−	−	−
SSU0685	phosphomethylpyrimidine kinase	+	+	+	+	+	+
SSU0686	hydroxyethylthiazole kinase	+	+	+	+	+	+
SSU0687	thiamine-phosphate pyrophosphorylase	+	+	+	+	+	+
SSU0706	muramidase-released protein precursor	−	−	+	−	−	−
SSU0722	putative L-threonine aldolase	+	+	+	−	−	−
SSU0829	plasmid addiction system, toxin protein	−	−	−	+	+	+
SSU0830	conserved hypothetical protein	−	−	−	+	+	+
SSU0879	zinc metalloproteinase	−	−	−	+	+	+
SSU0885	acetyltransferase (GNAT) family protein	−	−	−	−	+	+
SSU1036*	putative transposase	+	−	−	−	−	−
SSU1050	hyaluronidase precursor (pseudogene)	+	+	+	+	+	+
SSU1054	putative membrane protein	−	−	−	+	+	+
SSU1067	conserved hypothetical protein	+	+	+	−	−	−
SSU1074	putative membrane protein	+	−	−	−	−	−
SSU1126	mannosyl-glycoprotein endo-beta-N-acetylglucosaminidase (fragment)	+	−	+	−	−	−
SSU1163	conserved hypothetical protein	−	−	−	+	+	+
SSU1201	putative surface-anchored protein	−	−	−	−	+	+
SSU1242	VanZ like family protein	−	−	−	+	+	+
SSU1272	type I restriction-modification system S protein	−	−	−	+	−	−
SSU1273	type I restriction-modification system M protein	−	−	−	+	+	+
SSU1274	type I restriction-modification system R protein	−	−	−	+	+	+
SSU1383	putative acyl carrier protein phosphodiesterase	+	+	+	−	−	−
SSU1402	putative membrane protein	−	+	+	+	+	+
SSU1403	PadR-like family regulator protein	+	+	+	+	+	+
SSU1405*	putative transposase	−	−	−	+	−	−
SSU1422*	putative transposase	−	−	−	+	−	−
SSU1546	putative membrane protein	−	−	−	+	+	+
SSU1631*	putative transposase	−	−	+	−	−	−
SSU1718	putative membrane protein	+	+	+	−	−	−
SSU1773	putative surface-anchored serine protease	−	−	−	+	+	+
SSU1795	toxin-antitoxin system, toxin protein	+	−	−	+	+	+
SSU1796	toxin-antitoxin system, antitoxin protein	+	−	−	+	+	+
SSU1852	putative amino-acid ABC transporter permease protein	+	+	+	−	−	−
SSU1866	metal cation ABC transporter membrane protein	+	+	+	−	−	−
SSU1872	CAAX amino terminal protease family protein	+	+	+	−	−	−
SSU1881	sortase SrtD	−	−	−	+	−	−
SSU1882	sortase SrtC	+	+	+	+	−	−
SSU1883	sortase SrtB	+	−	+	+	−	−
SSU1885	putative surface-anchored protein	+	+	+	−	−	−
SSU1886	putative surface-anchored protein (pseudogene)	+	+	+	+	−	−
SSU1901	putative nucleotidase	−	+	−	−	−	−
SSU1934	putative exported protein	+	+	+	−	−	−

Orthologues identified using reciprocal Fasta, against the *S. pneumoniae* strains R6, TIGR4 and D39, and *S. agalactiae* strains NEM316, 2603V/R and A909. Orthologues with matches to *S. mutans* UA159, *S. gordonii* Challis CH1, *S. sanguinis* SK36, *S. pyogenes* Manfredo, *S. equi* 4047, *S. uberis* 0140J, or *S. thermophilus* CNRZ1066 were not included in the list. * contained on a putative MGE.

The final common CDS encodes a hyaluronidase. Hyaluronidase has been described as a virulence factor of *S. pneumoniae*; the addition of hyaluronidase to an intra-nasal inoculum was necessary to cause invasive disease in an infant mouse model of meningitis [99]. Hyaluronidase mutants do not appear to be attenuated in a murine model of meningitis after intracerebral infection [100], suggesting that this degradative enzyme may be important for the bacteria to cross the mucosa or the BBB during the early stages of pathogenesis. Notably in all five *S. suis* strains sequenced, including the human meningitis strains this CDS is present as a pseudogene. A study investigating the distribution of hyaluronidases in a diverse collection of *S. suis* isolates identified fewer than 30% exhibited hyaluronate lyase activity *in vitro*, although all the isolates tested (n. 309) contained the hyaluronidase gene (*hly*) [101]. In many cases mutations were identified that disrupted the expression of *hly*. Notably hyaluronate lyase activity was absent in the majority of isolates associated with invasive disease (meningitis and septicaemia), whereas activity was detected more often in isolates associated with pneumonia.

Given the unknown potential to cause meningitis of the *S. agalactiae* and *S. pneumoniae* strains sequenced, variably distributed matches should not be ruled out as having important roles in causing meningitis. Some of these CDSs encode functions that are important for pathogenesis, such as the zinc metalloproteinase previously mentioned. Orthologues of this protein are present in all of the *S. pneumoniae* strains compared but absent in *S. agalactiae* (Table 3). Components of the two pilus clusters are amongst the differentially distributed matches.

### Comparative genomics of two geographically distinct human disease strains SC84 and BM407

*S. suis* strain SC84 is a human isolate, and was isolated from the same outbreak as Chinese *S. suis* strain 05ZYH33 [8]. Strains from this outbreak have shown to belong to MLST sequence type 7 [7]. Strain P1/7 is MLST sequence type 1 and, as a single locus variant, belongs to the same clonal complex as SC84. Previous comparisons of strain P1/7 with *S. suis* strains 98HAH33 and 05ZYH33 showed that only 83.6% and 83.3% respectively of the P1/7 CDSs had orthologous matches. It was also reported that 4.9% of the P1/7 genome was unique in comparison to *S. suis* 05ZYH33 and 98HAH33 [7]. However, according to our analysis, all of the CDSs in the strain P1/7 genome have equivalent coding regions in the *S. suis* 05ZYH33 and 98HAH33 sequences. The difference in the number of orthologues identified can be accounted for by differences in gene prediction. The only large scale region of difference is associated with the 89 kb putative PI that is present in the two sequenced ST7 strains [7], and absent in P1/7.

The genome of *S. suis* strain SC84 consists of a single circular chromosome of 2,095,898 bp (accession number FM252031) and containing 1,985 CDSs (Table 1), of which 87 are pseudogenes or gene fragments (Table S1). Strain BM407 was isolated from a case of human meningitis in Vietnam in 2004 and, like strain P1/7, has MLST sequence type 1. The genome of *S. suis* strain BM407 consists of a single circular chromosome of 2,146,229 bp (accession number FM252032) containing 2,040 CDSs (Table 1) with 108 pseudogenes or gene fragments, and a plasmid (pBM407) of 24,579 bp, (accession number FM252033) which contains 18 CDSs with 3 pseudogenes or gene fragments (Table S1).

### Genome structure

**Figure 4:**
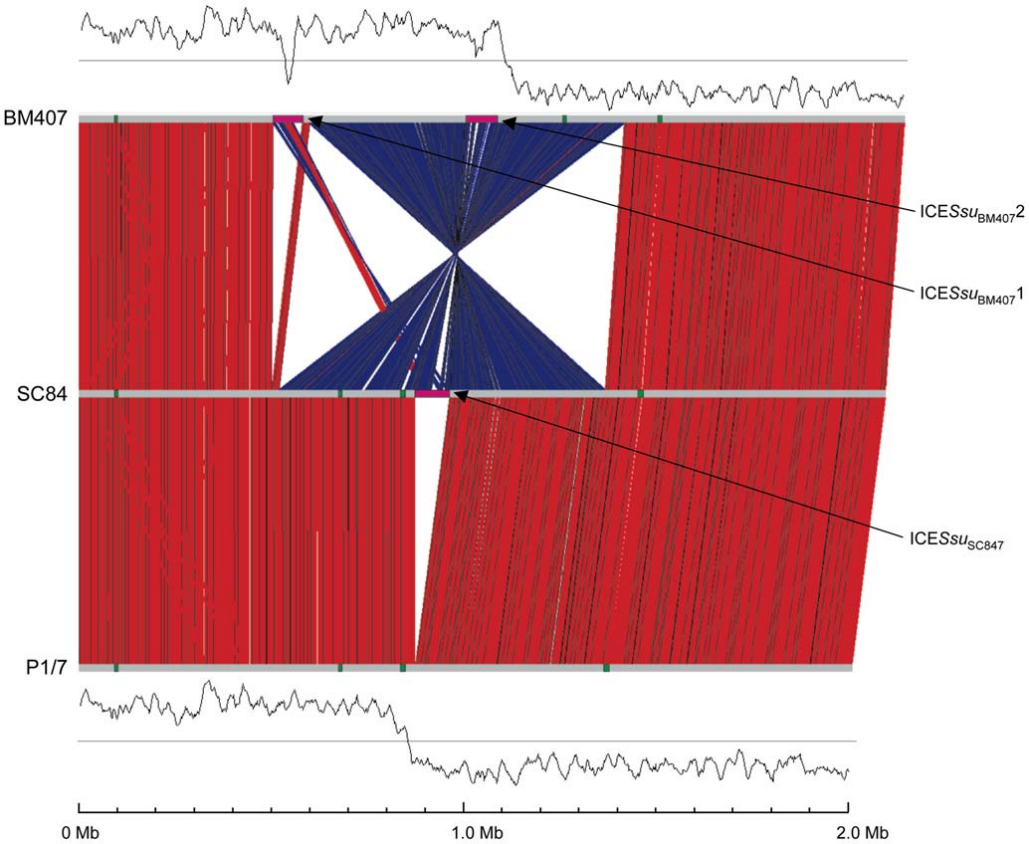
Chromosomal comparisons of *S. suis* strains. Pairwise comparisons of the chromosomes of *S. suis* strains P1/7, SC84 and BM407 displayed using the Artemis Comparison Tool (ACT) [26]. The sequences have been aligned from the predicted replication origins (*oriC*; right). The coloured bars separating sequences (red and blue) represent similarity matches identified by BLASTN analysis [15], with a score cut off of 100. Red lines link matches in the same orientation; blue lines link matches in the reverse orientation. The green coloured boxes on the horizontal grey lines mark up the extent of genomic islands identified on the chromosomes; pink boxes mark up the extent ICE regions.

The chromosomes of strains P1/7 and SC84 have a conserved structure and are collinear along their length (Figure 4). The BM407 chromosome is collinear, with the exception of a large inversion (nucleotide 600371 to 1416050). The rearrangement is due to recombination events between identical IS*3* elements on opposite replichores. Strains P1/7 and SC84 have asymmetric GC skews (Figure 4), whereas BM407 does not. Whilst the asymmetric conformation is present in two of the three strains sequenced, the BM407 conformation is likely to be the more common form of the *S. suis* chromosome as it maintains the replichore symmetry, placing the origin and terminus of replication at opposite ends of the circular chromosome, a common feature of bacterial circular chromosomes [102]. Screening a collection of Vietnamese *S. suis* ST1 strains [3] using PCR showed that the chromosome is found in alternative orientations in closely related strains, suggesting that the IS*3*-mediated recombination generating the chromosomal rearrangement is an ongoing process (data not shown).

Comparison of the three *S. suis* genomes reveals discrete regions of difference that distinguish these closely related strains. For strains P1/7 and SC84, the regions of difference are exclusively associated with MGEs; all of the genes identified in strain P1/7 outside MGEs have orthologues in strain SC84. Strain BM407 also contains variation associated with MGEs, but additionally there are two regions that appear to be deleted, resulting in a reduction in the number of genes on the chromosome of this strain in comparison to the other two. The first is a small region of 268 bp, flanked by 30 bp direct repeats, that encodes a putative membrane protein (in P1/7, SSU1245). The second absent region is 3.3 kb and contains 2 complete CDSs encoding subunits of an ATP-dependent exonuclease RexAB and the C-terminal region of Mrp (SSUBM407_1126; equivalent to bases 733785 to 743796 in strain P1/7). Immediately downstream of *mrp* is a partial IS*1216* transposase. The 3-prime region of *mrp* (pSSUBM407_006) is found on the plasmid pBM407, along with *rexAB* (pSSUBM407_015 and pSSUBM407_016), the genes for the missing chromosomal ATP-dependent exonuclease. The plasmid also contains three copies of IS*1216*, and it is likely that the chromosomal *mrp* and *rexAB* locus was deleted by IS*1216*-mediated recombination.

#### Small-scale variation

The three strains also exhibited small-scale variation at the nucleotide level including SNPs, insertions and deletions. Of the three strains BM407 appears to be the most diverse: comparison of strain BM407 to P1/7 identified 977 SNPs, 201 insertions, and 122 deletions in the core chromosome (excluding ICE regions); and comparison to SC84 identified 1017 SNPs, 232 insertions, and 133 deletions, whereas a comparison of strain P1/7 to SC84 identified 85 SNPs, 10 insertions, and 15 deletions.

The relative levels of variation are, on the face of it, at odds with the relatedness of the strains as defined by MLST: P1/7 belongs to the same sequence type as BM407, whereas SC84 is a single locus variant of P1/7. MLST is based on variation at only seven loci in the genome however, so it should not be surprising that it does not necessarily reflect the overall diversity at the whole genome level. Analysis of the distribution and density of SNPs in the genomes suggest that recombination has generated allelic variation at certain loci in the BM407 chromosome, thereby increasing the apparent level of diversity. Eight loci in the BM407 chromosome contained a high density of SNPs and were predicted by Clonalframe to be regions that have undergone recombination (Table 4). In total the SNPs in these regions account for ∼60% of the SNPs in the comparison with P1/7 and SC84.

**Table 4:** Putative regions of recombination identified in the BM407 genome.

CDS	Product	Coordinates	Number of SNPs
**Region 1**			
SSUBM407_0131	acetate kinase	124591..125778	62
SSUBM407_0132	membrane protein	126092..126643	48
SSUBM407_0133	folypolyglutamate synthase	126699..127955	48
SSUBM407_0134	putative glutamyl-aminopeptidase	129061..128000	19
**Region 2**			
SSUBM407_0646	putative surface-anchored zinc carboxypeptidase	715332..712147	33
SSUBM407_0647	putative cardiolipin synthetase	715548..717095	16
**Region 3**			
SSUBM407_0662	N-acetylmuramoyl-L-alanine amidase	730766..733573	41
**Region 4**			
SSUBM407_0668	polysaccharide export system ATP-binding protein	739785..740999	18
SSUBM407_0669	rhamnan synthesis protein F family protein	741020..742744	40
SSUBM407_0670	exopolysaccharide biosynthesis protein	742777..744036	16
**Region 5**			
SSUBM407_0840	sensor histidine kinase	742777..744036	37
**Region 6**			
SSUBM407_1445	glucan 1,6-alpha-glucosidase	1533333..1531717	16
SSUBM407_1446	sucrose phosphorylase	1534855..1533407	32
SSUBM407_1447	multiple sugar-binding transport system permease protein	1535755..1534925	33
SSUBM407_1448	multiple sugar-binding transport system permease protein	1536641..1535766	23
SSUBM407_1449	multiple sugar-binding protein precursor	1538008..1536773	16
**Region 7**			
SSUBM407_1489	conserved hypothetical protein	1572295..1571534	31
SSUBM407_1490	ABC-type glycine betaine transport system protein	1573816..1572305	38
**Region 8**			
SSUBM407_1989	acetyltransferase (GNAT) family protein	2097869..2097417	39
SSUBM407_1990	aldo/keto reductase family protein	2098790..2097879	14

SNP were identified in a comparison with the other ST 1 strain, P1/7.

Several of the putatively recombined loci in BM407 encode functions or proteins that potentially have a role in virulence or survival in the host. Two of the loci encode components of transport systems, that include an ABC-type glycine betaine transport system protein (SSUBM407_1490) homologous (56.1% identity at the amino acid level) to a bile exclusion system protein BilEB from *Listeria monocytogenes* shown to have a role in virulence [103]; and a binding protein-dependent transport system (SSUBM407_1447 to SSUBM407_1449) orthologous to a system from *S. mutans* responsible for the uptake of melibiose, raffinose, and isomaltotriose and the metabolism of melibiose, sucrose, and isomaltosaccharides [104]. One of the loci is composed of three CDSs that are part of the putative exopolysaccharide biosynthesis cluster previously described. Two of the loci contain cell wall anchored surface proteins, encoding a zinc carboxypeptidase (SSUBM407_0646) and a N-acetylmuramoyl-L-alanine amidase (SSUBM407_0662). A single regulatory CDS (SSUBM407_0840) is found in the loci that appear to have undergone recombination. This CDS encodes a sensor kinase which is part of a two component regulatory system operon, with the SNPs being found in the N-terminal region of the sensor kinase protein. SSUBM407_0840 is homologous to *ciaH* of *S. pneumoniae* (49.3% identity at the amino acid level) which plays a role in antibiotics resistance and transformation competence [105]. Mutations within this regulatory protein increase resistance to the cephalosporin cefotaxime.

There are fewer SNPs in the comparisons between P1/7 and SC84 than were described in the publication by Chen *et al.* (2007) which compared 98HAH33, 05ZYH33 and P1/7. Interestingly, a comparison of SC84 with 05ZYH33, both strains from the same outbreak, identified 2602 SNPs, 596 insertions, and 304 deletions. This is in marked contrast to the low number of SNPs identified in the comparison between P1/7 and SC84, two strains isolated from different hosts, on different continents over 20 years apart.

Strains from the Sichuan outbreak have been found to be ST7 [8]. Examination of the sequence at MLST loci of 05ZYH33 revealed that they do not match the expected alleles for those of ST7. In comparison to SC84, which itself belongs to ST7, SNPs were identified at two of the seven MLST loci in the 05ZYH33 sequence: the *aroA* locus has 29 SNPs, and *gki* has 2 SNPs. Although, it is possible that 05ZYH33 is actually a different sequence type, albeit a double locus variant of ST7. The predicted sequence type of 98HAH33 is more divergent still, being a triple locus variant of ST7; the *aroA* has 6 SNPs, *cpn* has 1 single base deletion, and *dpr* has 1 single base deletion. The most likely explanation for these observations is that the sequence data for the 05ZYH33 and 98HAH33 strains is incorrect.

#### Mobile genetic elements

Four regions of the P1/7 genome were identified that had properties indicative of recent acquisition, which include 1 prophage-like region and 3 miscellaneous islands, which have been designated genomic islands. None of these genomic islands carried characterized or putative virulence factors. Equivalent genomic islands are present in the SC84 genome. BM407 contains three of the genomic islands present in P1/7, but is lacking genomic island 3 (in strain P1/7 nucleotides 835135 to 846313; Figure 4). In contrast to some streptococcal species such as *S. pyogenes* [106], the proportion of the genome comprised by MGEs is relatively small, at 1.8% of the P1/7 genome, suggesting that the pan-genome of this species is smaller than other streptococci [96], [107], and that there may be some limitations or restrictions to the amount of recent horizontal gene transfer in this species.

Previous sequencing of the two *S. suis* strains associated with meningitis outbreaks in China in 1998 and 2005 identified highly similar 89 kb regions present in both genomes which were designated a candidate PI [7]. This region is absent in strain P1/7, but strain SC84 contains an almost identical region. This island has a composite structure, contains several regions that appear to be integrated MGEs and has a structure similar to integrative conjugative elements (ICE) found in other streptococci and other bacteria [108]. Accordingly this region has been designated ICE*Ssu*_SC84_.

#### Integrative conjugative elements

ICE are commonly found in streptococcal genomes. The most common are Tn*916*-type elements, which almost invariably carry the tetracycline resistance determinant *tetM*, often accompanied by other drug resistance genes, and Tn*5252*-type elements, which are larger and more variable in sequence. In many cases, Tn*5252* transposons are carrying Tn*916* elements; varying arrangements of the two are observed in different genomes, suggesting this association has arisen independently on a number of occasions. Analogous with the bacterial chromosome as a whole, the ICE have core and accessory components to their gene complement. The conjugative machinery appears to be relatively well conserved between the ICE, with all of the streptococcal Tn*5252*-like elements sharing a large recombination protein, as well as VirB4-type and VirD4-type proteins. Furthermore, a genus-wide comparison of streptococcal ICE reveals a surprising level of similarity in their complement of cargo genes. The *pezAT* addiction toxin system, first identified in the chromosome of *S. pneumoniae*, is found on elements present in *S. suis*, *S. pneumoniae* and *S. agalactiae*, presumably aiding fixation of the transposon following integration. Bacteriocins, either alone or as part of a gene cluster encoding the accompanying processing machinery, are present on all the sequenced *S. suis* and *S. pneumoniae* [*109*] elements. Many of the other cargo coding sequences appear to be involved in increasing the stress tolerance of the host: *abi* genes are common, and the ICE of *S. dysgalactiae* subsp. *equisimilis* and *S. agalactiae* 2603V/R encode multiple heavy metal resistance genes. Of greater clinical importance, sequenced streptococcal ICE can carry genes for resistance to tetracycline, erythromycin, chloramphenicol, trimethoprim, aminoglycosides and streptomycin.

**Figure 5:**
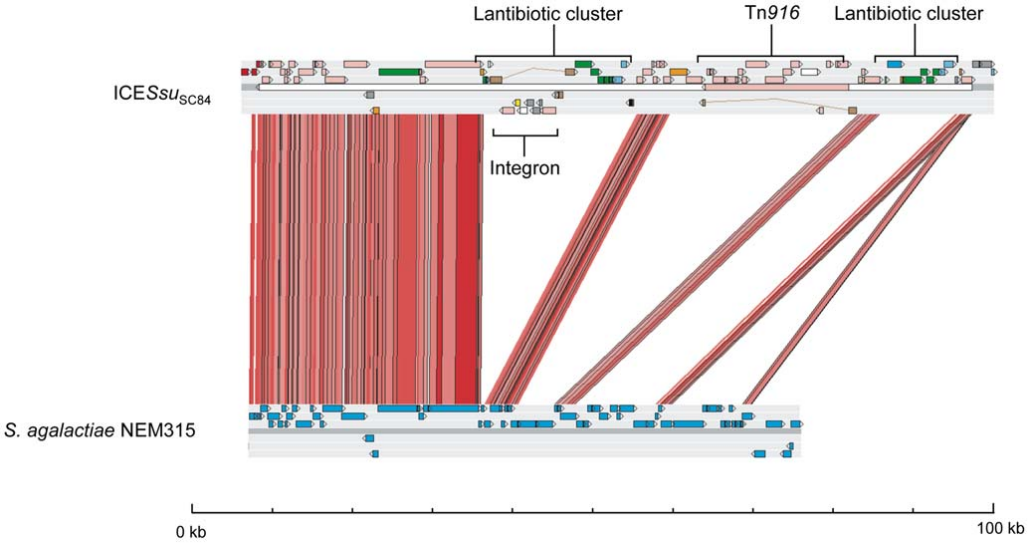
Comparisons of the ICE regions from *S. suis* strains SC84 and *S. agalactiae* NEM315. The results of a TBLASTX comparison of the ICE*Ssu*_SC84_ from strain SC84 and *S. agalactiae* NEM315 Tn*2424* is displayed using the Artemis Comparison Tool (ACT) [26].

ICE*Ssu*_SC84_ is integrated into the SC84 *S. suis* genome in the 3-prime region of the gene encoding the 50S ribosomal protein L7/L12 (SSUSC84_0891). ICE*Ssu*_SC84_ has extended similarity to a Tn*2424* region (Figure 5) found in *S. agalactiae* strain NEM316 [94], with three regions of difference. The first of these contains a putative bacteriocin biosynthesis cluster that is disrupted by a putative integron carrying five CDSs including an aminoglycoside resistance gene (aminoglycoside 6-adenylyltansferase; SSUSC84_0863). The second contains MGE-associated CDSs, including a Tn*916* insertion that carries a tetracycline resistance gene (*tetM*; SSUSC84_0827). The third region contains genes associated with putative lantibiotic export/resistance and an asparagine synthetase. The only component of the 89kb island that is an obvious candidate for a pathogenicity determinant is a surface-anchored protein that contains a LPXTG motif that is annotated as an agglutinin receptor (SSUSC84_0873). This protein is found in the *S. agalactiae* element (and other streptococci) and contains a glucan-binding domain.

**Figure 6:**
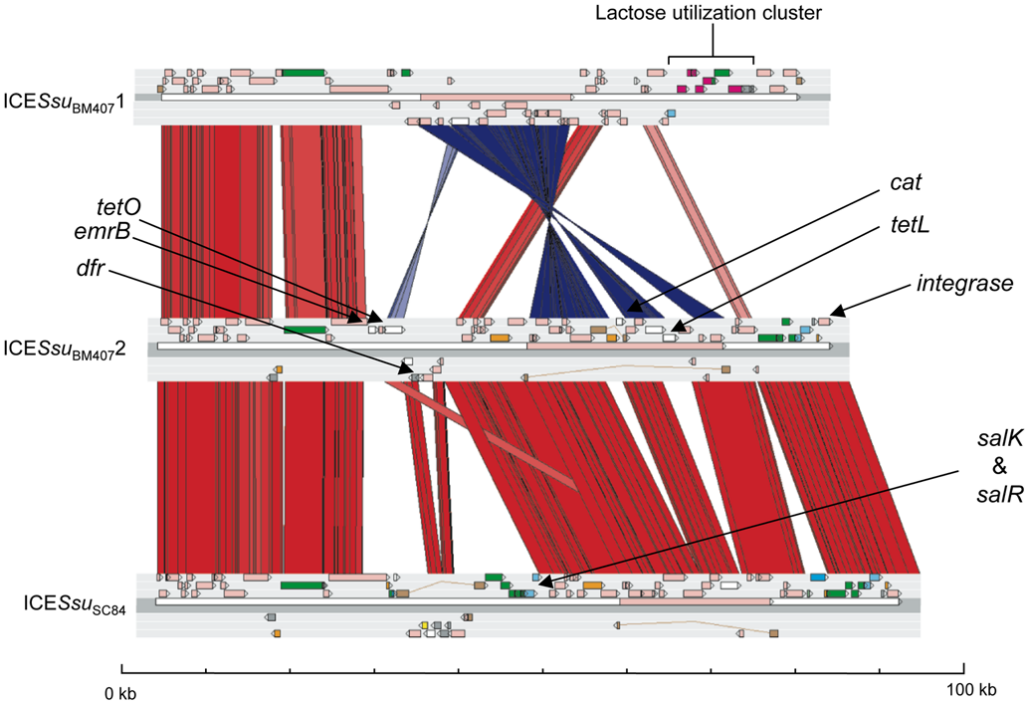
Comparisons of the ICE regions from *S. suis* strains BM407 and SC84. The results of a TBLASTX comparison of the ICE*Ssu*_BM407_1 and ICE*Ssu*_BM407_2 regions from strain BM407, and ICE*Ssu*_SC84_ from strain SC84 is displayed using the Artemis Comparison Tool (ACT) [26].

The BM407 genome contains two regions with extended similarity to ICE*Ssu*_SC84_. The first of these, ICE*Ssu*_BM407_1, is inserted into a luciferase-like monooxygenase (SSUBM407_0455), resulting in the disruption of this CDS. The second, ICE*Ssu*_BM407_2, is found at the same locus as ICE*Ssu*_SC84_; the element is inserted into the 3-prime region of the gene encoding the 50S ribosomal protein L7/L12 (SSUBM407_0933). Although ICE*Ssu*_BM407_2 and ICE*Ssu*_SC84_ are inserted at the same attachment site it is likely that they have arisen in these two strains by independent insertion events. Not only do the two elements contain regions of difference, the conserved DNA in the ICEs is more diverse than the core chromosome DNA (the ICEs exhibit ∼95−98% identity, in contrast to ∼99−100% identity for chromosomal DNA). ICE*Ssu*_BM407_2 and ICE*Ssu*_SC84_ have conserved tyrosine family site-specific integrases (SSUBM407_1003 and SSUSC84_0807; Figure 6), which is probably the explanation for why these elements integrated at the same site in the respective chromosomes, whereas ICE*Ssu*_BM407_1 contains a serine family recombinase (SSUBM407_0524) that directs the insertion of this element elsewhere on the BM407 chromosome.

Comparison of the BM407 and SC84 ICEs shows that CDSs associated with conjugation are conserved (Figure 6). The ICEs contain regions of sequence divergence with ICE*Ssu*_BM407_1 appearing to be most diverse, containing a greater proportion of unique sequence, and an inverted region. All three elements contain variants of Tn*916*; ICE*Ssu*_BM407_1 and ICE*Ssu*_SC84_ have Tn*916* with the same gene content, whilst ICE*Ssu*_BM407_2 contains additional genes encoding the tetracycline resistance protein TetL (SSUBM407_0984) and chloramphenicol acetyltransferase (SSUBM407_0980). In addition to the drug resistance genes associated with Tn*916*, ICE*Ssu*_BM407_2 also carries genes encoding the tetracycline resistance protein TetO (SSUBM407_0954), erythromycin ribosome methylase ErmB (SSUBM407_0952) and dihydrofolate reductase (SSUBM407_0957), in a region of difference located elsewhere on the element.

Unlike ICE*Ssu*_BM407_2, ICE*Ssu*_BM407_1 does not carry as many CDSs associated with drug resistance. Amongst its cargo are a lactose utilization operon (SSUBM407_0510 to SSUBM407_0519), including a glucokinase-like protein, a galactose-6-phosphate isomerase, a tagatose-6-phosphate kinase, a tagatose 1,6-diphosphate aldolase, a 6-phospho-beta-galactosidase, and a PTS system lactose-specific transporter, which are absent in ICE*Ssu*_BM407_2 and ICE*Ssu*_SC84_. The chromosome of BM407 (and P1/7 and SC84) already contains homologues of these CDSs at two loci (SSUBM407_0879 to SSUBM407_0889 and SSUBM407_1060). Interestingly the chromosomal copy of the tagatose-6-phosphate kinase *lacC* (SSUBM407_0883) is mutated in BM407, therefore the provision of an additional copy of this CDS on ICE*Ssu*_SC84_ complements the mutation. Complete lactose utilization operons have been found on other ICEs, including the *S. agalactiae* NEM315 ICE.

Contained on ICE*Ssu*_SC84_ is a two component regulator, *salK*/*salR* (SSUSC84_0849 and SSUSC84_0850), that has been implicated in the virulence of the Chinese isolates in an animal model [110]. These regulatory components are part of a larger operon encoding lantibiotic biosynthesis, similar to the salivaricin cluster of *Streptococcus salivarius* [111]. Neither of the ICEs in BM407 contains this cluster (Figure 6); these elements contain alternative cargo CDSs in equivalent regions on these elements. Upstream of the operon is a CDS encoding a lantibiotic precursor (SSUSC84_0866). In SC84, and also in 05ZYH33 and 98HAH33, the operon has been disrupted by the insertion of a 7.9 kb element containing 9 CDSs, one of which encodes an aminoglycoside 6-adenylyltransferase (SSUSC84_0863), into the homologue of the *S. salivarius* salivaricin C biosynthesis gene *salM* (SSUSC84_0856). The precise regulatory function of SalK/SalR remains unclear, but their operonic association with the lantibiotic cluster suggests that their primary role is associated with this function. However it is possible that regulatory components of this operon may have been adapted to other functions. *S. pyogenes* with a mutation in *salY*, a gene encoding a putative ABC transporter permease of a lantibiotic operon, was attenuated for virulence in a zebrafish invasive-disease model [112]. Analysis of the *S. pyogenes* lantibiotic cluster showed that it contained a homologue of *salM* that was mutated, suggesting that like the lantibiotic cluster in *S. suis* SC84, this cluster also no longer produced a functional lantibiotic, but it was still was essential for full virulence.

Screening of 91 Vietnamese *S. suis* serotype 2 strains of MLST ST1 isolated from patients with meningitis, for the presence of ICE*Ssu*_SC84_ using PCR targeted at the insertion sites [7], indicated that this ICE is not absolutely required for virulence in humans as 16 (17.6%) strains lacked the element (manuscript in preparation). In addition, only 41 (45%) carried sequences homologous to the *salK/salR* genes, suggesting that the presence of these genes cannot be used as a marker of virulence. Similar to strains BM407 and SC84, all strains containing ICE-associated sequences were resistant to tetracycline whereas those that were negative in the screening were sensitive, indicating the common presence of ICE carrying *tet* genes in these isolates.

## Discussion

The sequencing and detailed annotation of the *S. suis* P1/7, SC84 and BM407 genomes provides valuable data for studying the evolutionary events that are shaping the virulence and drug resistance in this emerging zoonotic pathogen.

There is strong evidence that genetic background influences the ability of *S. suis* to cause zoonotic infections as human disease isolates are almost always serotype 2 and are very closely related by MLST. Of the 121 STs in the *S. suis* MLST data base (http://ssuis.mlst.net/), only five STs have representative isolated from humans, and four (ST1, ST6, ST7 and ST84) out of the five are all part of the same clonal complex. Two of the strains in this study (P1/7 and BM407) introduce a greater degree of diversity into the comparisons, insofar as that they are from different geographical areas, and belong to different STs.

A major feature of the previously sequenced *S. suis* strains' genomes hypothesised to be important for pathogenicity, is an 89 kb ICE region designated as a candidate PI [7]. BM407 also contains an almost identical ICE with some notable differences that encompass drug resistance genes, and a two-component regulatory system associated with a bacteriocin cluster. Sequence variation in the BM407 ICE suggest that although it is present at an orthologous site, it has been independently acquired.

The comparative analysis suggests that ICEs have been the major contributor to the evolution of drug resistance in the strains of *S. suis* examined. Although it is not possible to pinpoint the origins of the *S. suis* ICEs, due to the limited number of these elements in the databases, it is apparent from the analyses that they are closely related to ICEs found in phylogenetically distant streptococci. It is worth noting that some of these species occupy the same niches as *S. suis*, suggesting that inter-species genetic exchange is taking place. This has implications for defining the pan-genomes of some streptococcal species, since it may not be possible to define their boundaries if the pool of MGEs they exchange is effectively communal.

Aside from the ICEs, the virulence determinant inventories of P1/7, SC84 and BM407 strains are identical. Small nucleotide differences resulting from point mutation and recombination were observed in the genome comparisons. This variation may have subtle effects on the expression and function of these genes, and as a corollary, generate differences in the virulence phenotypes. To this extent it is possible that some of the small nucleotide differences observed in the SC84 and BM407 genomes in comparison to P1/7 may influence the ability of these strains to cause disease. However, as the capacity of P1/7 to cause human disease is unknown, it is not possible to speculate on the impact of genomic differences identified in this study on host-association. Future studies intending to increase the depth of sequencing in this zoonotic lineage will help unravel the pathogenic significance of sequence variation.

## Supporting Information

Table S1 Pseudogenes in the genome of S. suis strains P1/7, SC84 and BM407.
(0.15 MB DOC)

Table S2 Streptococcus suis orphan CDSs in the in the genome of S. suis strain P1/7.
(0.24 MB DOC)

Figure S1 Rhamnose-based polysaccharide cluster of strain P1/7 A) Structure and functional organisation of the Rhamnose-based polysaccharide cluster of strain P1/7. B) Comparison of the Rhamnose-based polysaccharide cluster from S. suis P1/7 with the RGP rml and rgp gene clusters of Streptococcus mutans UA159. The comparison of the RGP clusters from the S. suis P1/7 (top) and S. mutans UA159 and (bottom) is displayed using the Artemis Comparison Tool (ACT) [26]. The red bars separating each genome represent similarity matches identified by TBLASTX analysis [14].
(0.35 MB PDF)
