# Supplementary material for: Rapid Evolution of Virulence and Drug Resistance in the Emerging Zoonotic Pathogen Streptococcus suis
Source: PLoS One. 2009 Jul 15;4(7):e6072. doi: 10.1371/journal.pone.0006072 (PMC2705793; doi:10.1371/journal.pone.0006072)
Supplement: Table S1 — Pseudogenes in the genome of S. suis strains P1/7, SC84 and BM407. (0.15 MB DOC) [file pone.0006072.s001.doc]

**Table S1. Pseudogenes and partial genes in the *S. suis* genomes.**

| **Product** | **P1/7** | **SC84** | **BM407** |
| --- | --- | --- | --- |
| hypothetical protein (pseudogene) | Intact | SSUSC840035 | Intact |
| putative competence-specific global transcription modulator (fragment) | SSU0068 | SSUSC840064 | SSUBM4070064 |
| putative phosphoribosylaminoimidazole carboxylase (fragment) | SSU0087 | SSUSC840083 | SSUBM4070083 |
| putative integrase (fragment) | SSU0111 | SSUSC840106 | SSUBM4070106 |
| putative phosphoribosylaminoimidazole carboxylase (fragment) | SSU0121A | Absent | SSUBM4070117 |
| conserved hypothetical protein (fragment) | SSU0121B | SSUSC840116a | SSUBM4070118 |
| putative microcin immunity protein (pseudogene) | SSU0125 | SSUSC840120 | SSUBM4070122 |
| putative exported protein (pseudogene) | Intact | Intact | SSUBM4070129 |
| putative surface-anchored protein (pseudogene) | SSU0171 | SSUSC840164 | SSUBM4070166 |
| putative beta-lactamase (pseudogene) | SSU0189 | SSUSC840181 | SSUBM4070183 |
| copper-transporting P-type ATPase CopA (fragment) | SSU0207 | SSUSC840198 | SSUBM4070200 |
| probable thiol peroxidase (fragment) | SSU0207A | SSUSC840199 | SSUBM4070201 |
| putative surface-anchored protein (pseudogene) | SSU0254 | SSUSC840243 | SSUBM4070245 |
| transposase (fragment) | SSU0254A | SSUSC840244 | SSUBM4070246 |
| integrase (fragment) | SSU0256 | SSUSC840245 | SSUBM4070247 |
| ABC transporter ATP-binding membrane protein (pseudogene) | Intact | Intact | SSUBM4070254 |
| haloacid dehalogenase-like hydrolase (pseudogene) | SSU0316 | SSUSC840304 | SSUBM4070306 |
| isochorismatase family protein (pseudogene) | SSU0321 | SSUSC840308 | SSUBM4070310 |
| acetyltransferase (GNAT) family (pseudogene) | SSU0339 | SSUSC840326 | SSUBM4070329 |
| isochorismatase family protein (pseudogene) | SSU0341 | SSUSC840327 | SSUBM4070330 |
| conserved hypothetical protein (pseudogene) | SSU0365 | SSUSC840351 | SSUBM4070354 |
| putative valine-tRNA ligase (fragment) | SSU0414 | SSUSC840399 | SSUBM4070402 |
| accessory pilus subunit (pseudogene) | SSU0425 | SSUSC840410 | SSUBM4070413 |
| transposase (fragment) | SSU0429 | SSUSC840413 | SSUBM4070416 |
| putative transposase (fragment) | SSU0452 | SSUSC840436 | SSUBM4070439 |
| conserved hypothetical protein (fragment) | SSU0454 | SSUSC840438 | SSUBM4070441 |
| flavin monooxygenase (pseudogene) | Intact | Intact | SSUBM4070455 |
| haloacid dehalogenase-like hydrolase (pseudogene) | Intact | Intact | SSUBM4070540 |
| putative transposase (fragment) | SSU0503A | SSUSC840488 | SSUBM4071310 |
| putative transposase (fragment) | SSU0505A | SSUSC840489 | SSUBM4071309 |
| putative transposase (fragment) | SSU0505B | SSUSC840490 | SSUBM4071308 |
| N-acylneuraminate cytidylyltransferase (fragment) | SSU0530 | SSUSC840514 | SSUBM4071283 |
| transposase (pseudogene) | SSU0539 | SSUSC840521 | SSUBM4071276 |
| putative transposase (fragment) | SSU0543 | SSUSC840524 | SSUBM4071273 |
| putative transposase (pseudogene) | SSU0545 | SSUSC840526 | SSUBM4071271 |
| putative transposase (fragment) | SSU0549 | SSUSC840527 | SSUBM4071270 |
| putative transposase (fragment) | SSU0550 | SSUSC840528 | SSUBM4071269 |
| putative transposase (fragment) | SSU0551 | SSUSC840529 | SSUBM4071268 |
| putative tyrosine recombinase (fragment) | SSU0552 | SSUSC840530 | SSUBM4071267 |
| D-alanine--D-alanine ligase (pseudogene) | SSU0554 | SSUSC840531 | SSUBM4071265 |
| hypothetical protein (pseudogene) | Intact | Intact | SSUBM4071266 |
| putative transposase (fragment) | SSU0562 | SSUSC840538 | SSUBM4071259 |
| peptidase family M20/M25/M40 protein (pseudogene) | Intact | Intact | SSUBM4071237 |
| putative transposase (fragment) | SSU0612 | SSUSC840585 | SSUBM4071212 |
| putative membrane protein (pseudogene) | SSU0630 | SSUSC840603 | SSUBM4071194 |
| type III restriction-modification system, modification enzyme (fragment) | SSU0640 | SSUSC840608 | SSUBM4071189 |
| putative transposase (pseudogene) | SSU0643 | SSUSC840610 | SSUBM4071187 |
| type I restriction-modification system, modification protein (pseudogene) | SSU0652 | SSUSC840619 | SSUBM4071178 |
| conserved hypothetical protein (pseudogene) | SSU0658 | SSUSC840625 | SSUBM4071172 |
| DeoR family regulatory protein (fragment) | Intact | Intact | SSUBM4071169 |
| putative phospholipase/carboxylesterase (pseudogene) | SSU0679 | SSUSC840645 | SSUBM4071152 |
| muramidase-released protein precursor (fragment) | Intact | Intact | SSUBM4071126 |
| topoisomerase IV subunit B (fragment) | SSU0712 | SSUSC840677 | SSUBM4071121 |
| Fic protein family protein (pseudogene) | SSU0713 | SSUSC840678 | SSUBM4071120 |
| conserved hypothetical protein (fragment) | SSU0729A | SSUSC840694 | SSUBM4071104 |
| transposase (fragment) | SSU0756 | SSUSC840720 | SSUBM4071078 |
| cell envelope proteinase (pseudogene) | Intact | SSUSC840721 * | SSUBM4071077 * |
| putative multidrug resistance protein (pseudogene) | SSU0761 | SSUSC840725 | SSUBM4071073 |
| tunicamycin resistance protein (fragment) | SSU0822 | SSUSC840785 | SSUBM4071025 |
| abortive infection bacteriophage resistance related protein (pseudogene) | SSU0823 | SSUSC840786 | SSUBM4071024 * |
| putative ATP-binding protein (pseudogene) | Absent | SSUSC840822 | SSUBM4070990 |
| putative DNA-binding protein (fragment) | Absent |  | SSUBM4070986 |
| putative membrane protein (pseudogene) | Absent | Intact | SSUBM4070978 |
| transposase (fragment) | Absent | SSUSC840848 | SSUBM4070961a |
| two-component sensor histidine kinase (fragment) | Absent | Intact | SSUBM4070960c |
| putative membrane protein (fragment) | Absent | Intact | SSUBM4070960b |
| DNA recombinase (fragment) | Absent | Intact | SSUBM4070960a |
| adenine phosphoribosyltransferase (fragment) | Absent | Intact | SSUBM4070954a |
| hypothetical protein (fragment) | Absent | Absent | SSUBM4070951a |
| putative DNA methylase (fragment) | Absent | Intact | SSUBM4070951 |
| lantibiotic modifying enzyme (pseudogene) | Absent | SSUSC840856 | Absent |
| DNA recombinase (fragment) | Absent | SSUSC840857 | Absent |
| glucose-1-phosphate adenylyltransferase (pseudogene) | SSU0871 | Intact | SSUBM4070907 |
| UvrABC system protein B (fragment) | SSU0880 | SSUSC840925 | SSUBM4070899 |
| tagatose-6-phosphate kinase (pseudogene) | SSU0896 | SSUSC840941 | SSUBM4070883 |
| putative integrase (pseudogene) | SSU0904 | SSUSC840948 | SSUBM4070876 |
| conserved hypothetical protein (pseudogene) | SSU0928 | SSUSC840971 | SSUBM4070853 |
| putative lipoprotein (pseudogene) | Intact | Intact | SSUBM4070841 |
| conserved hypothetical protein (pseudogene) | SSU0961 | SSUSC841001 | SSUBM4070823 |
| conserved hypothetical protein (pseudogene) | SSU0963 | SSUSC841002 | SSUBM4070822 |
| acetyltransferase (GNAT) family protein (pseudogene) | SSU0965 | SSUSC841004 | SSUBM4070818 |
| conserved hypothetical protein (pseudogene) | Intact | Intact | SSUBM4070794 |
| UDP-N-acetylglucosamine 1-carboxyvinyltransferase 1 (pseudogene) | Intact | Intact | SSUBM4070775 |
| hyaluronate lyase (pseudogene) | SSU1050 | SSUSC841087 | SSUBM4070736 |
| mannosyl-glycoprotein endo-beta-N-acetylglucosaminidase (fragment) | SSU1126 | SSUSC841159 | SSUBM4070663 |
| putative transposase (fragment) | SSU1152A | SSUSC841185 | SSUBM4070637 |
| putative transposase (fragment) | SSU1152B | SSUSC841186 | SSUBM4070636 |
| glyoxalase/bleomycin resistance protein/dioxygenase superfamily protein (pseudogene) | Intact | Intact | SSUBM4070614 |
| transposase (pseudogene) | SSU1249 | SSUSC841281 | SSUBM4071328 |
| transposase (pseudogene) | SSU1250 | SSUSC841282 | SSUBM4071329 |
| VanZ like family protein (pseudogene) | SSU1264A | SSUSC841294 | SSUBM4071341 |
| putative glycogen phosphorylase (pseudogene) | Intact | SSUSC841295 | Intact |
| type I restriction-modification system S protein (fragment) | SSU1271 | SSUSC841301 | SSUBM4071348 |
| putative esterase (pseudogene) | Intact | Intact | SSUBM4071378 |
| putative membrane protein (fragment) | SSU1388 | SSUSC841418 | SSUBM4071465 |
| putative RNA binding protein (fragment) | SSU1426 | SSUSC841455 | SSUBM4071502 |
| conserved hypothetical protein (pseudogene) | SSU1446 | SSUSC841475 | SSUBM4071522 |
| putative IS200-like transposase (pseudogene) | SSU1450 | SSUSC841479 | SSUBM4071526 |
| serum opacity factor (pseudogene) | SSU1474 | SSUSC841502 | SSUBM4071549 |
| putative NAPDPH-flavin oxidoreductase (pseudogene) | SSU1535 | SSUSC841561 | SSUBM4071609 |
| CAAX amino terminal protease family protein (pseudogene) | SSU1593 | SSUSC841619 | SSUBM4071667 |
| putative transposase (pseudogene) | SSU1658 | SSUSC841683 | SSUBM4071731 |
| 30S ribosomal protein S15 (fragment) | SSU1667 | SSUSC841691 | SSUBM4071739 |
| hypothetical protein (pseudogene) | SSU1689 | SSUSC841713 | SSUBM4071761 |
| 50S ribosomal protein L13 (fragment) | SSU1693 | SSUSC841716 | SSUBM4071764 |
| conserved hypothetical protein (pseudogene) | SSU1724 | SSUSC841746 | SSUBM4071794 |
| conserved hypothetical protein (fragment) | SSU1734 | SSUSC841756 | SSUBM4071804 |
| 3-isopropylmalate dehydratase large subunit | Intact | Intact | SSUBM4071809 |
| abortive infection protein (fragment) | SSU1872A | SSUSC841895 | SSUBM4071943 |
| response regulator protein (fragment) | SSU1873 | SSUSC841896 | SSUBM4071944 |
| sortase SrtB (pseudogene) | Intact | Intact | SSUBM4071952 |
| major pilus subunit (pseudogene) | SSU1886 | SSUSC841906 | SSUBM4071954 |

Orthologues where present are presented in the same row. The systematic ID of mutated genes are indicated. Where orthologues in the other strains are not mutated they are listed as intact. Where orthologues are not present in the other strains they indicated as absent. * indicates pseudogenes contain alternative mutations.
