# Supplementary material for: Rapid Evolution of Virulence and Drug Resistance in the Emerging Zoonotic Pathogen Streptococcus suis
Source: PLoS One. 2009 Jul 15;4(7):e6072. doi: 10.1371/journal.pone.0006072 (PMC2705793; doi:10.1371/journal.pone.0006072)
Supplement: Table S2 — Streptococcus suis orphan CDSs in the in the genome of S. suis strain P1/7. (0.24 MB DOC) [file pone.0006072.s002.doc]

**Table S2. *Streptococcus suis* orphan CDSs in the genome of *S. suis* strain P1/7.**

| **ID** | **Product** |
| --- | --- |
| SSU0035 | hypothetical protein |
| SSU0036 | hypothetical protein |
| SSU0039 | putative membrane protein |
| SSU0040 | CAAX amino terminal protease family protein |
| SSU0041 | putative membrane protein |
| SSU0043 | putative membrane protein |
| SSU0044 | ABC transporter ATP-binding membrane protein |
| SSU0051 | transposase |
| SSU0052 | hypothetical protein |
| SSU0053 | transposase |
| SSU0055 | hypothetical protein |
| SSU0059 | hypothetical protein |
| SSU0068 | putative competence-specific global transcription modulator (fragment) |
| SSU0084 | hypothetical protein |
| SSU0086 | hypothetical protein |
| SSU0087 | putative phosphoribosylaminoimidazole carboxylase (fragment) |
| SSU0111 | putative integrase (fragment) |
| SSU0117 | hypothetical protein |
| SSU0121A | putative phosphoribosylaminoimidazole carboxylase (fragment) |
| SSU0121B | conserved hypothetical protein (fragment) |
| SSU0145 | putative membrane protein |
| SSU0152 | putative endopeptidase |
| SSU0171 | putative surface-anchored protein (pseudogene) |
| SSU0186 | putative surface-anchored protein |
| SSU0195 | putative tagatose-6-phosphate kinase |
| SSU0196 | HhH-GPD superfamily base excision DNA repair protein |
| SSU0197 | hypothetical protein |
| SSU0201 | putative surface-anchored protein |
| SSU0202 | putative exported protein |
| SSU0206 | glucosamine-6-phosphate isomerase |
| SSU0207 | copper-transporting P-type ATPase CopA (fragment) |
| SSU0207A | probable thiol peroxidase (fragment) |
| SSU0224 | AMP-binding enzyme |
| SSU0233 | hypothetical protein |
| SSU0252 | hypothetical protein |
| SSU0253 | putative surface-anchored protein |
| SSU0254 | putative surface-anchored protein (pseudogene) |
| SSU0254A | transposase (fragment) |
| SSU0256 | integrase (fragment) |
| SSU0287 | putative membrane protein |
| SSU0296 | hypothetical protein |
| SSU0297 | putative transcription regulation protein |
| SSU0302 | putative transcription regulation protein |
| SSU0304 | putative lipase |
| SSU0307 | putative membrane protein |
| SSU0327 | putative deoxyguanosinetriphosphate triphosphohydrolase |
| SSU0333 | hypothetical protein |
| SSU0339 | acetyltransferase (GNAT) family (pseudogene) |
| SSU0350 | conserved hypothetical protein |
| SSU0355 | GntR family regulatory protein |
| SSU0363 | hypothetical protein |
| SSU0365 | conserved hypothetical protein (pseudogene) |
| SSU0413 | putative membrane protein |
| SSU0414 | putative valine-tRNA ligase (fragment) |
| SSU0416 | DEAD/DEAH box family helicase |
| SSU0423 | hypothetical protein |
| SSU0424 | putative signal peptidase I 2 |
| SSU0425 | putative accessory pilus subunit (pseudogene) |
| SSU0428 | sortase |
| SSU0429 | transposase (fragment) |
| SSU0449 | hypothetical protein |
| SSU0450 | putative signal peptidase I 3 |
| SSU0451 | putative membrane protein |
| SSU0454 | conserved hypothetical protein (fragment) |
| SSU0459 | hypothetical protein |
| SSU0469 | hypothetical protein |
| SSU0488 | putative transposase |
| SSU0496 | putative membrane anchored protein |
| SSU0503A | putative transposase (fragment) |
| SSU0505A | putative transposase (fragment) |
| SSU0511 | TetR family regulatory proteins |
| SSU0520 | putative rhamnosyl transferase |
| SSU0522 | hypothetical protein |
| SSU0523 | putative membrane protein |
| SSU0525 | ptative glycosyltransferase |
| SSU0527 | putative membrane protein |
| SSU0528 | putative membrane protein |
| SSU0529 | putative membrane protein |
| SSU0530 | N-acylneuraminate cytidylyltransferase (fragment) |
| SSU0539 | transposase (pseudogene) |
| SSU0541 | putative transposase |
| SSU0542 | transposase |
| SSU0543 | putative transposase (fragment) |
| SSU0545 | putative transposase (pseudogene) |
| SSU0549 | putative transposase (fragment) |
| SSU0550 | putative transposase (fragment) |
| SSU0551 | putative transposase (fragment) |
| SSU0552 | putative tyrosine recombinase (fragment) |
| SSU0554 | D-alanine--D-alanine ligase (pseudogene) |
| SSU0556 | hypothetical protein |
| SSU0562 | putative transposase (fragment) |
| SSU0563 | UDP-galactopyranose mutase |
| SSU0567 | putative transposase |
| SSU0571 | conserved hypothetical protein |
| SSU0572 | putative plasmid addiction system, toxin protein |
| SSU0573 | multi antimicrobial extrusion (MATE) family transporter |
| SSU0587 | glycosyl hydrolase family protein |
| SSU0592 | putative exported protein |
| SSU0593 | putative exported protein |
| SSU0594 | putative exported protein |
| SSU0595 | putative exported protein |
| SSU0612 | putative transposase (fragment) |
| SSU0630 | putative membrane protein (pseudogene) |
| SSU0632 | putative membrane protein |
| SSU0634 | hypothetical protein |
| SSU0635 | hypothetical protein |
| SSU0640 | type III restriction-modification system, modification enzyme (fragment) |
| SSU0642 | type III restriction-modification system, restriction enzyme |
| SSU0643 | putative transposase (pseudogene) |
| SSU0669 | hypothetical protein |
| SSU0684 | putative phosphatase |
| SSU0689 | putative uridine phosphorylase |
| SSU0692 | ABC transporter ATP-binding protein |
| SSU0696 | putative phage holin protein |
| SSU0712 | topoisomerase IV subunit B (fragment) |
| SSU0715 | putative membrane protein |
| SSU0716 | hypothetical protein |
| SSU0723 | putative membrane protein |
| SSU0729A | conserved hypothetical protein (fragment) |
| SSU0756 | transposase (fragment) |
| SSU0774 | putative membrane protein |
| SSU0776 | putative membrane protein |
| SSU0777 | putative membrane protein |
| SSU0784 | putative membrane protein |
| SSU0787 | putative membrane protein |
| SSU0788 | hypothetical protein |
| SSU0798 | putative lipoprotein |
| SSU0799 | hypothetical protein |
| SSU0800 | hypothetical protein |
| SSU0801 | putative lipoprotein |
| SSU0802 | putative restriction enzyme |
| SSU0803 | putative restriction enzyme modulator protein |
| SSU0822 | tunicamycin resistance protein (fragment) |
| SSU0826 | phosphoglucomutase/phosphomannomutase family protein |
| SSU0836 | putative lipoprotein |
| SSU0837 | hypothetical protein |
| SSU0844 | haloacid dehalogenase-like hydrolase |
| SSU0859 | Maf-like protein |
| SSU0865 | conserved hypothetical protein |
| SSU0880 | UvrABC system protein B (fragment) |
| SSU0881 | hypothetical protein |
| SSU0888 | putative membrane protein |
| SSU0889 | hypothetical protein |
| SSU0900 | hypothetical protein |
| SSU0902 | hypothetical protein |
| SSU0903 | putative DNA-binding protein |
| SSU0904 | putative integrase (pseudogene) |
| SSU0906 | hypothetical protein |
| SSU0907 | hypothetical protein |
| SSU0928 | conserved hypothetical protein (pseudogene) |
| SSU0946 | putative exported protein |
| SSU0947 | putative transposase |
| SSU0961 | conserved hypothetical protein (pseudogene) |
| SSU0966 | putative transposase |
| SSU0967 | putative transposase |
| SSU0970 | putative membrane protein |
| SSU0971 | conserved hypothetical protein |
| SSU0994 | conserved hypothetical protein |
| SSU1026 | conserved hypothetical protein |
| SSU1045 | hypothetical protein |
| SSU1049 | hypothetical protein |
| SSU1060 | hypothetical protein |
| SSU1065 | transposase |
| SSU1069 | putative membrane protein |
| SSU1073 | putative membrane protein |
| SSU1075 | putative membrane protein |
| SSU1080 | hypothetical protein |
| SSU1081 | putative membrane protein |
| SSU1094 | putative exported protein |
| SSU1115 | putative glycosyl transferase |
| SSU1125 | putative membrane protein |
| SSU1128 | putative surface-anchored protein |
| SSU1140 | putative membrane protein |
| SSU1152A | putative transposase (fragment) |
| SSU1152B | putative transposase (fragment) |
| SSU1176 | putative membrane protein |
| SSU1187 | ABC transporter ATP-biding protein |
| SSU1188 | ABC transporter ATP-biding protein |
| SSU1197 | conserved hypothetical protein |
| SSU1207 | putative lipoprotein |
| SSU1232 | haloacid dehalogenase-like hydrolase |
| SSU1233 | ABC transporter ATP-binding membrane protein |
| SSU1243 | putative membrane protein |
| SSU1244 | hypothetical protein |
| SSU1245 | putative membrane protein |
| SSU1247 | putative transposase |
| SSU1250 | transposase (pseudogene) |
| SSU1271 | type I restriction-modification system S protein (fragment) |
| SSU1277 | hypothetical protein |
| SSU1280 | putative membrane protein |
| SSU1282 | acetyltransferase (GNAT) family protein |
| SSU1285 | hypothetical protein |
| SSU1286 | hypothetical protein |
| SSU1287 | putative exported protein |
| SSU1297 | hypothetical protein |
| SSU1303 | putative lipoprotein |
| SSU1324 | putative transposase |
| SSU1377 | putative membrane protein |
| SSU1378 | putative membrane protein |
| SSU1379 | putative membrane protein |
| SSU1382 | putative membrane protein |
| SSU1388 | putative membrane protein (fragment) |
| SSU1399 | hypothetical protein |
| SSU1404 | putative membrane protein |
| SSU1423 | putative transposase |
| SSU1424 | hypothetical protein |
| SSU1426 | putative RNA binding protein (fragment) |
| SSU1437 | hypothetical protein |
| SSU1450 | putative IS200-like transposase (pseudogene) |
| SSU1455 | putative transposase |
| SSU1457 | putative transposase |
| SSU1474 | serum opacity factor (pseudogene) |
| SSU1476 | putative surface-anchored protein |
| SSU1477 | hypothetical protein |
| SSU1479 | putative oligopeptidase |
| SSU1508 | hypothetical protein |
| SSU1532 | putative membrane protein |
| SSU1533 | putative membrane protein |
| SSU1552 | putative membrane protein |
| SSU1553 | putative membrane protein |
| SSU1554 | putative membrane protein |
| SSU1555 | putative membrane protein |
| SSU1556 | putative membrane protein |
| SSU1557 | putative exported protein |
| SSU1559 | putative thiamine biosynthesis lipoprotein |
| SSU1562 | pyridine nucleotide-disulphide oxidoreductase family protein |
| SSU1563 | putative heptaprenyl diphosphate synthase protein |
| SSU1564 | putative membrane protein |
| SSU1572 | conserved hypothetical protein |
| SSU1591 | conserved hypothetical protein |
| SSU1612 | hypothetical protein |
| SSU1616 | putative exported protein |
| SSU1633 | putative membrane protein |
| SSU1640 | putative lipoprotein |
| SSU1658 | putative transposase (pseudogene) |
| SSU1667 | 30S ribosomal protein S15 (fragment) |
| SSU1687 | conserved hypothetical protein |
| SSU1689 | hypothetical protein (pseudogene) |
| SSU1693 | 50S ribosomal protein L13 (fragment) |
| SSU1697 | putative membrane protein |
| SSU1698 | putative membrane protein |
| SSU1700 | putative membrane protein |
| SSU1706 | putative transposase |
| SSU1724 | conserved hypothetical protein (pseudogene) |
| SSU1725 | sugar phosphotransferase system (PTS), IIBC component |
| SSU1726 | conserved hypothetical protein |
| SSU1730 | hypothetical protein |
| SSU1731 | putative membrane protein |
| SSU1734 | conserved hypothetical protein (fragment) |
| SSU1745 | LemA family protein |
| SSU1769 | hypothetical protein |
| SSU1788 | radical SAM superfamily protein |
| SSU1790 | hypothetical protein |
| SSU1800 | hypothetical protein |
| SSU1811 | glyoxalase/bleomycin resistance protein/dioxygenase superfamily protein |
| SSU1816 | Major Facilitator Superfamily protein |
| SSU1833 | hypothetical protein |
| SSU1837 | hypothetical protein |
| SSU1849 | putative surface-anchored amylopullulanase |
| SSU1850 | LacI family regulatory protein |
| SSU1854 | putative amidase |
| SSU1868 | putative membrane protein |
| SSU1871 | putative membrane protein |
| SSU1872A | CAAX amino terminal protease family protein (fragment) |
| SSU1875 | putative membrane protein |
| SSU1876 | putative membrane protein |
| SSU1888 | putative accessory pilus subunit |
| SSU1889 | putative accessory pilus subunit |
| SSU1890 | putative membrane protein |
| SSU1892 | putative membrane protein |
| SSU1897 | conserved hypothetical protein |
| SSU1901 | putative nucleotidase |
| SSU1904 | putative transposase |
| SSU1905 | putative membrane protein |
| SSU1911 | putative choline binding protein |
| SSU1913 | putative membrane protein |
| SSU1926 | glycosyl transferase |
| SSU1927 | putative beta-glucosidase |
| SSU1928 | putative membrane protein |
| SSU1929 | putative beta-glucosidase |
| SSU1932 | Calcineurin-like phosphoesterase |
| SSU1933 | putative fumarate reductase flavoprotein subunit |
| SSU1936 | hypothetical protein |
| SSU1946 | conserved hypothetical protein |
