# Supplementary figures and images for: Rapid Evolution of Virulence and Drug Resistance in the Emerging Zoonotic Pathogen Streptococcus suis
Source: PLoS One. 2009 Jul 15;4(7):e6072. doi: 10.1371/journal.pone.0006072 (PMC2705793; doi:10.1371/journal.pone.0006072)

A

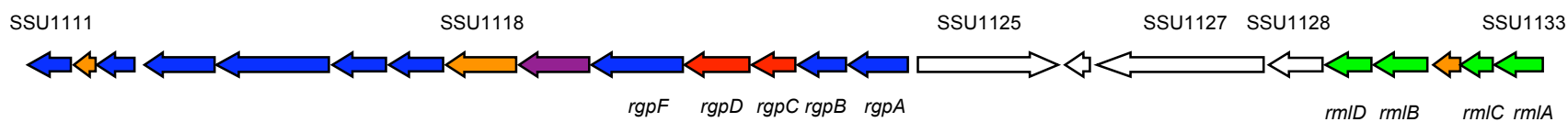

B

*S. suis* P1/7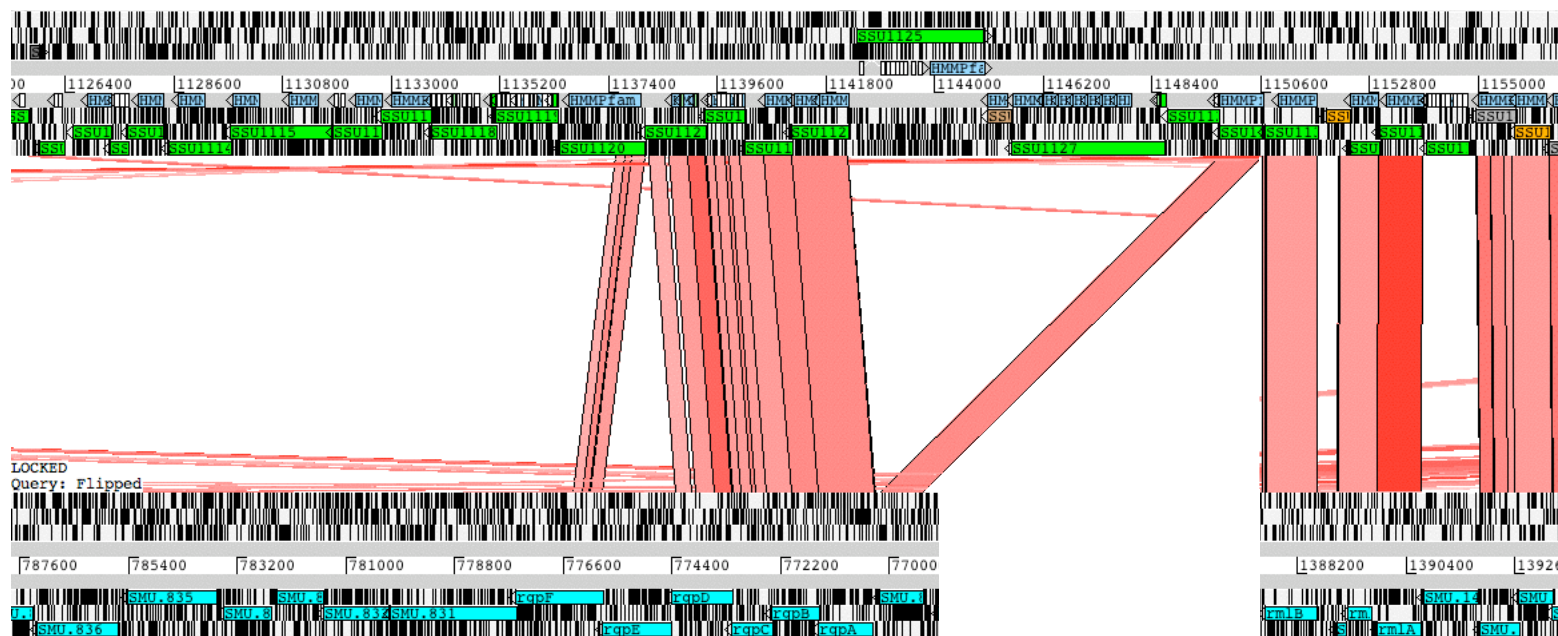

Supplement: Figure S1 — Rhamnose-based polysaccharide cluster of strain P1/7 A) Structure and functional organisation of the Rhamnose-based polysaccharide cluster of strain P1/7. B) Comparison of the Rhamnose-based polysaccharide cluster from S. suis P1/7 with the RGP rml and rgp gene clusters of Streptococcus mutans UA159. The comparison of the RGP clusters from the S. suis P1/7 (top) and S. mutans UA159 and (bottom) is displayed using the Artemis Comparison Tool (ACT) [26]. The red bars separating each genome represent similarity matches identified by TBLASTX analysis [14]. (0.35 MB PDF) [file pone.0006072.s003.pdf]
